# Association between aerobic fitness and the functional connectome in patients with schizophrenia

**DOI:** 10.1007/s00406-022-01411-x

**Published:** 2022-04-30

**Authors:** Lukas Roell, Isabel Maurus, Daniel Keeser, Temmuz Karali, Boris Papazov, Alkomiet Hasan, Andrea Schmitt, Irina Papazova, Moritz Lembeck, Dusan Hirjak, Eliska Sykorova, Cristina E. Thieme, Susanne Muenz, Valentina Seitz, David Greska, Mattia Campana, Elias Wagner, Lisa Loehrs, Sophia Stoecklein, Birgit Ertl-Wagner, Johannes Poemsl, Astrid Roeh, Berend Malchow, Katriona Keller-Varady, Andreas Meyer-Lindenberg, Peter Falkai

**Affiliations:** 1 Department of Psychiatry and Psychotherapy, University Hospital, LMU Munich, Nussbaumstrasse 7, 80336 Munich, Germany; 2 Department of Radiology, University Hospital, LMU Munich, Munich, Germany; 3 Department of Psychiatry and Psychosomatics of the University Augsburg, Bezirkskrankenhaus Augsburg, University of Augsburg, Augsburg, Germany; 4 Laboratory of Neuroscience (LIM27), Institute of Psychiatry, University of Sao Paulo, São Paulo, Brazil; 5 Central Institute of Mental Health, Medical Faculty Mannheim, Heidelberg University, Heidelberg, Germany; 6 Department of Psychiatry and Psychotherapy, Medical Faculty, Technical University of Munich, University Hospital ‘Klinikum Rechts Der Isar’, Munich, Germany; 7 Department of Psychiatry and Psychotherapy, University Hospital Göttingen, Göttingen, Germany; 8 Hannover Medical School, Institute of Sports Medicine, Hannover, Germany; 9 NeuroImaging Core Unit Munich (NICUM), University Hospital LMU, Munich, Germany; 10 Division of Neuroradiology, Department of Diagnostic Imaging, The Hospital for Sick Children, Toronto, Canada

**Keywords:** Schizophrenia, Exercise, Fitness, Neuroimaging, fMRI, Functional connectivity

## Abstract

**Background:**

Schizophrenia is accompanied by widespread alterations in static functional connectivity associated with symptom severity and cognitive deficits. Improvements in aerobic fitness have been demonstrated to ameliorate symptomatology and cognition in people with schizophrenia, but the intermediary role of macroscale connectivity patterns remains unknown.

**Objective:**

Therefore, we aim to explore the relation between aerobic fitness and the functional connectome in individuals with schizophrenia. Further, we investigate clinical and cognitive relevance of the identified fitness-connectivity links.

**Methods:**

Patients diagnosed with schizophrenia were included in this cross-sectional resting-state fMRI analysis. Multilevel Bayesian partial correlations between aerobic fitness and functional connections across the whole brain as well as between static functional connectivity patterns and clinical and cognitive outcome were performed. Preliminary causal inferences were enabled based on mediation analyses.

**Results:**

Static functional connectivity between the subcortical nuclei and the cerebellum as well as between temporal seeds mediated the attenuating relation between aerobic fitness and total symptom severity. Functional connections between cerebellar seeds affected the positive link between aerobic fitness and global cognition, while the functional interplay between central and limbic seeds drove the beneficial association between aerobic fitness and emotion recognition.

**Conclusion:**

The current study provides first insights into the interactions between aerobic fitness, the functional connectome and clinical and cognitive outcome in people with schizophrenia, but causal interpretations are preliminary. Further interventional aerobic exercise studies are needed to replicate the current findings and to enable conclusive causal inferences.

**Trial registration:**

The study which the manuscript is based on is registered in the International Clinical Trials Database (ClinicalTrials.gov identifier [NCT number]: NCT03466112) and in the German Clinical Trials Register (DRKS-ID: DRKS00009804).

**Supplementary Information:**

The online version contains supplementary material available at 10.1007/s00406-022-01411-x.

## Introduction

Schizophrenia is described as a disorder of dysconnectivity characterized by deficits in synaptic functioning and myelination [1]. Those micro-scale alterations lead to impairments within neural macro-scale circuits which in turn drive psychopathological symptoms and cognitive deficits [2]. In recent years, various large-scale resting-state functional magnetic resonance imaging (fMRI) examinations have confirmed abnormalities of static functional connectivity (FC) patterns in severe mental disorders [3–8]. In resting-state fMRI, FC is defined as the temporal similarity of the blood-oxygen-level-dependent (BOLD) signal of two brain regions during rest and quantifies the degree of their connectedness [9–11]. Compared to healthy controls, hypo- and hyperconnectivities within and between core intrinsic connectivity networks (ICNs) represent typical functional deviations across multiple psychiatric conditions (e.g., schizophrenia, depression, anxiety disorder) [3–7]. Current large-scale evidence identifies schizophrenia-specific functional disconnections of particular seed regions in the salience network (SN), default-mode network (DMN), fronto-parietal network (FPN) and the limbic network [3].

ICNs are generally associated with essential aspects of human behavior like cognition, emotion, perception, interoception and action [12]. Correspondingly, across different psychiatric disorders FC alterations within and between the DMN, SN and FPN are related to deficits in different components of neurocognition such as inhibition control, fluid intelligence, spatial orientation or alertness [5]. In patients with schizophrenia, negative symptom severity is related to FC alterations within the DMN [6], while the cognitive domains of processing speed and working memory performance reveal associations with FC within the SN, the auditory network, the sensorimotor network and the visual network [7]. Both, negative symptoms and cognitive deficits in schizophrenia, remain difficult to treat using antipsychotic medication [13, 14], persist over the long term in most of the patients [15] and contribute to poor social and occupational functioning [16, 17] as well as to low recovery rates [18].

In healthy individuals, compelling evidence demonstrates that moderate exercise ameliorates general health [19] and improves cognitive functioning [20], while aerobic fitness is linked to widespread adaptations of FC across the whole brain [21]. In people with schizophrenia, different kinds of exercise treatments show small to medium beneficial effects on positive [22–26] and negative symptom severity [22–25, 27, 28], depressive symptoms [24, 29], several cognitive domains [25, 26, 29, 30], quality of life [24, 29] and global functioning [24, 26, 31]. In particular, exercise interventions aiming at enhancing aerobic fitness represent promising adjunctive treatment strategies in schizophrenia [22, 26, 30, 32].

Beneficial effects of such interventions are assumed to be mediated by multiple neurophysiological processes such as structural plasticity changes (e.g., increases in grey and white matter volumes) and molecular adaptations (e.g., changes in growth factor and neurotransmitter concentrations) [33, 34]. However, the mechanistic role of changes in macro-scale FC patterns that potentially drive the beneficial effects of aerobic exercise on psychiatric symptoms and cognition in schizophrenia has been neglected yet. Importantly, even the general association between aerobic fitness and global FC patterns in patients with schizophrenia has not been studied to date. Consequently, we do not know to which particular functional connections aerobic fitness is generally linked in people with schizophrenia and thus cannot derive hypotheses on behaviorally relevant, regional FC adaptations induced by aerobic exercise interventions.

The current cross-sectional study addresses this gap to enable hypothesis-driven aerobic exercise intervention approaches that investigate the mediating role of region-specific changes in FC. We aim to provide first insights into potential FC mechanisms that drive the beneficial link between aerobic fitness and psychopathological outcome. Therefore, we examine the relation between aerobic fitness and multiple functional connections across the whole brain (defined as the functional connectome) in patients with schizophrenia using a global, exploratory approach. Further, we investigate if those functional connections associated with aerobic fitness also demonstrate clinical or cognitive relevance and mediate the association between aerobic fitness and psychiatric symptoms and cognition.

## Methods

The ESPRIT C3 study is a clinical, randomized-controlled, multicenter trial examining the effects of an aerobic exercise intervention on multiple health outcomes in people with schizophrenia [35]. All patients were diagnosed with schizophrenia in accordance with DSM-IV and the majority received antipsychotic mediation. For inclusion and exclusion criteria as well as other study details see Maurus et al. [35]. The current cross-sectional investigation utilized the baseline data of the ESPRIT C3 study prior to intervention onset.

### Study sample

A sample of 101 patients with schizophrenia recruited at study centers LMU Hospital in Munich and Central Institute of Mental Health in Mannheim underwent MRI scans (for sample characteristics see Table 1). In case of four subjects, inclusion and exclusion criteria were not fulfilled although MRI data were available. Nine subjects had no resting-state fMRI sequences, while further nine subjects were excluded due to lacking image quality (suppl. S2). Depending on the corresponding statistical analysis (“Statistical data analysis”), a different number of subjects was included: regarding the correlations between aerobic fitness and the functional connectome, further 21 subjects had to be excluded because no fitness data were available resulting in 58 individuals considered in this analysis. With respect to the correlations between the functional connectome and clinical and cognitive scores, different amounts of participants had to be excluded depending on the number of invalid or missing values in each cognitive test battery resulting in 72 to 79 individuals included in this analysis (suppl. S6). In the context of the mediation analyses, the aforementioned 21 subjects were removed due to lacking fitness data. The number of missing values differed between clinical and cognitive tests leading to 51–58 patients included in this approach (suppl. S6).

**Table 1:** Sample characteristics

Attribute	*n*	Mean ± SD
Sample		
Full	101	–
After exclusion of screening failures	97	–
After exclusion of subjects with lacking or noisy fMRI sequences	79	–
After exclusion of subjects with lacking fitness data	58	–
Age (years)	97	37.35 ± 12.20
Sex		–
Male	62 (63.9%)	–
Female	35 (36.1%)	–
Site		
Munich	72 (74.2%)	–
Mannheim	25 (25.8%)	–
Chlorpromazine equivalents (CPZ)	97	372.11 ± 245.74
Years of education	97	14.48 ± 4.14
Disorder duration (years)	97	9.29 ± 9.24
Body-mass-index (BMI)	97	28.30 ± 5.31

Important sample characteristics of the whole sample are listed. All numbers relate to the study sample after exclusion of screening failures. Screening failures reflect cases that were removed due to inclusion and exclusion criteria. Data from other sub-samples are neglected because they are fairly similar

### Operationalization of aerobic fitness

Subjects performed a stepwise lactate threshold test on a stationary bicycle ergometer. A function describing the relation between wattage and lactate concentration was estimated. Lactate concentrations at around 2 mmol/l are supposed to represent the aerobic threshold [36]. We identified the individual aerobic threshold at lactate concentrations between 1.8 and 2.5 mmol/l according to the previously defined exercise protocol [35]. Achieved wattage at a subject-specific lactate concentration within this range divided by body weight represents individuals’ performance capability at an aerobic exercise intensity. We refer to this value using the term aerobic fitness.

### fMRI data acquisition and pre-processing

MRI data at both study centers were acquired in a whole-body 3.0 Tesla MRI Scanner (Magnetom Skyra, Siemens Healthcare, Erlangen, Germany). Subjects at both study sites underwent at least one echo-planar imaging (EPI) sequence and one T1-weighted magnetization prepared rapid gradient echo (MP-RAGE) sequence (see suppl. S1 for scanning parameters). Raw data files from the scanners were converted from DICOM to NIFTI format using dcm2niix software [37]. NIFTI files were embedded into a BIDS data structure [38].

Quality control was performed utilizing the automated software MRIQC [39]. Pre-processing was done using FMRIPREP [40] (suppl. S3). Within FMRIPREP, automatic removal of motion artifacts based on independent component analysis (ICA-AROMA) was utilized to extract aggressive noise regressors [41]. Framewise displacement [42, 43] and DVARS [43] as well as the temporal signal-to-noise ratio calculated via *fslmaths* from FSL v 6.0.4 [44] were evaluated again after pre-processing. The *slicer* attribute from NiBabel v3.2.1 [45] was administered to remove the first ten dummy scans of every pre-processed fMRI file. Images were smoothed (FWHM = 6 mm) with the *smooth_img* function from Nilearn v0.8.0 which was built on scikit-learn [46] and the temporal signal-to-noise ratio was checked again. Confound regression, detrending, low- and high-pass filtering (0.008–0.1 Hz) and signal standardization were performed within one step utilizing the *clean_img* function from Nilearn v0.8.0. Global signal, cerebrospinal fluid, white matter and the extracted noise components from ICA-AROMA were regressed from BOLD timeseries according to current findings on different denoising strategies [47].

### fMRI data post-processing

**Fig. 1:**
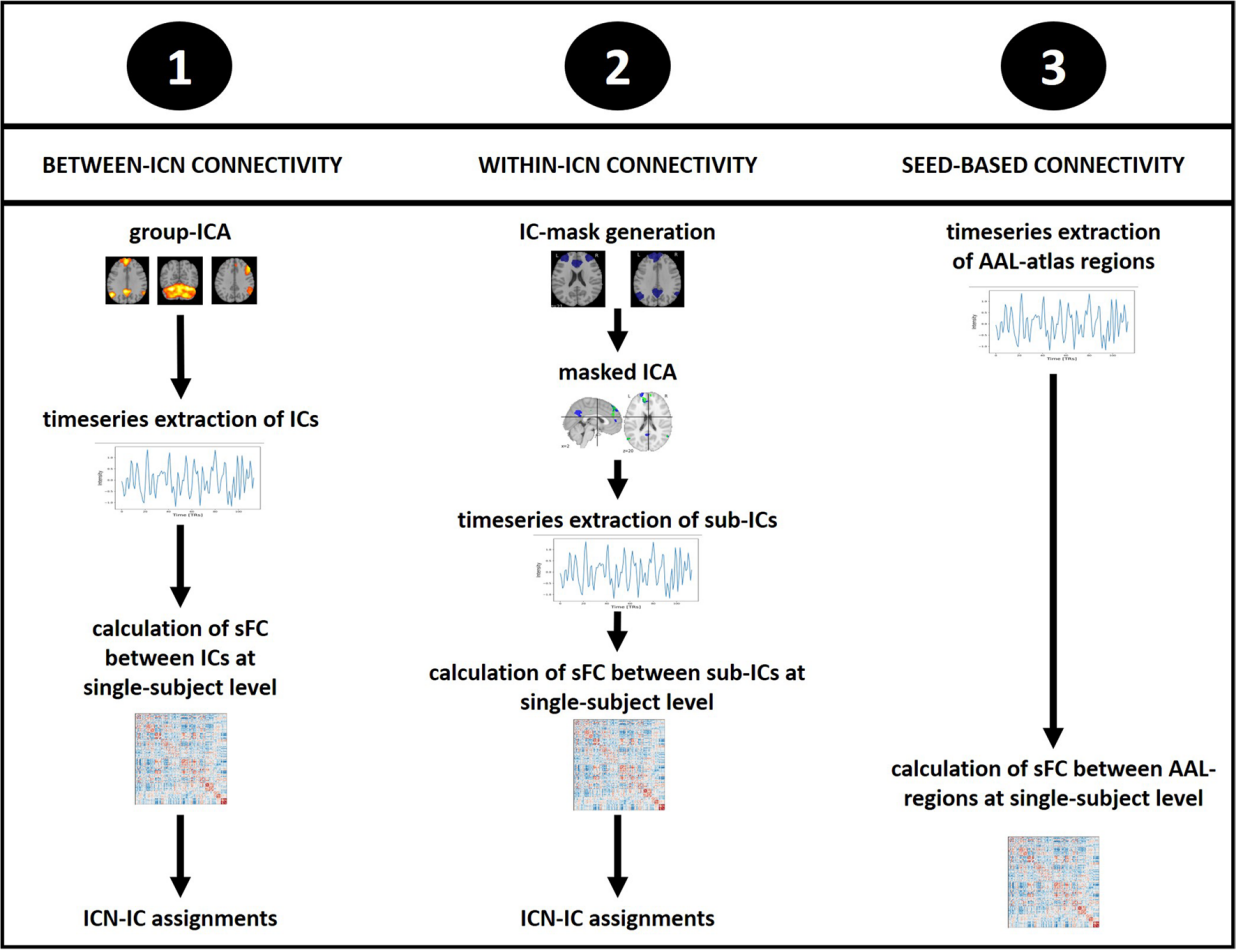
Workflow of the three resting-state fMRI analysis approaches. The analysis steps of the three fMRI approaches that aim to examine the functional connectome from different perspectives are visualized. Details on the workflows are described in the manuscript text

To explore the functional connectome in patients with schizophrenia, three different analyses were applied on denoised fMRI data (Fig. 1): (1) computation of FC between ICNs, (2) assessment of FC within ICNs and (3) a seed-based examination of FC between different brain regions. These approaches were selected to cover different perspectives on the broad concept of the functional connectome. While the first and second analysis provide insights into the functional organization of the human brain based on widespread ICNs, the seed-based approach takes into account the anatomical organization of the brain with a higher spatial resolution. Since we examine associations between aerobic fitness and the functional connectome from a global and exploratory perspective, we aimed to target multiple facets of the functional connectome. All three approaches are common in neuropsychiatric fMRI research.

**Fig. 2:**
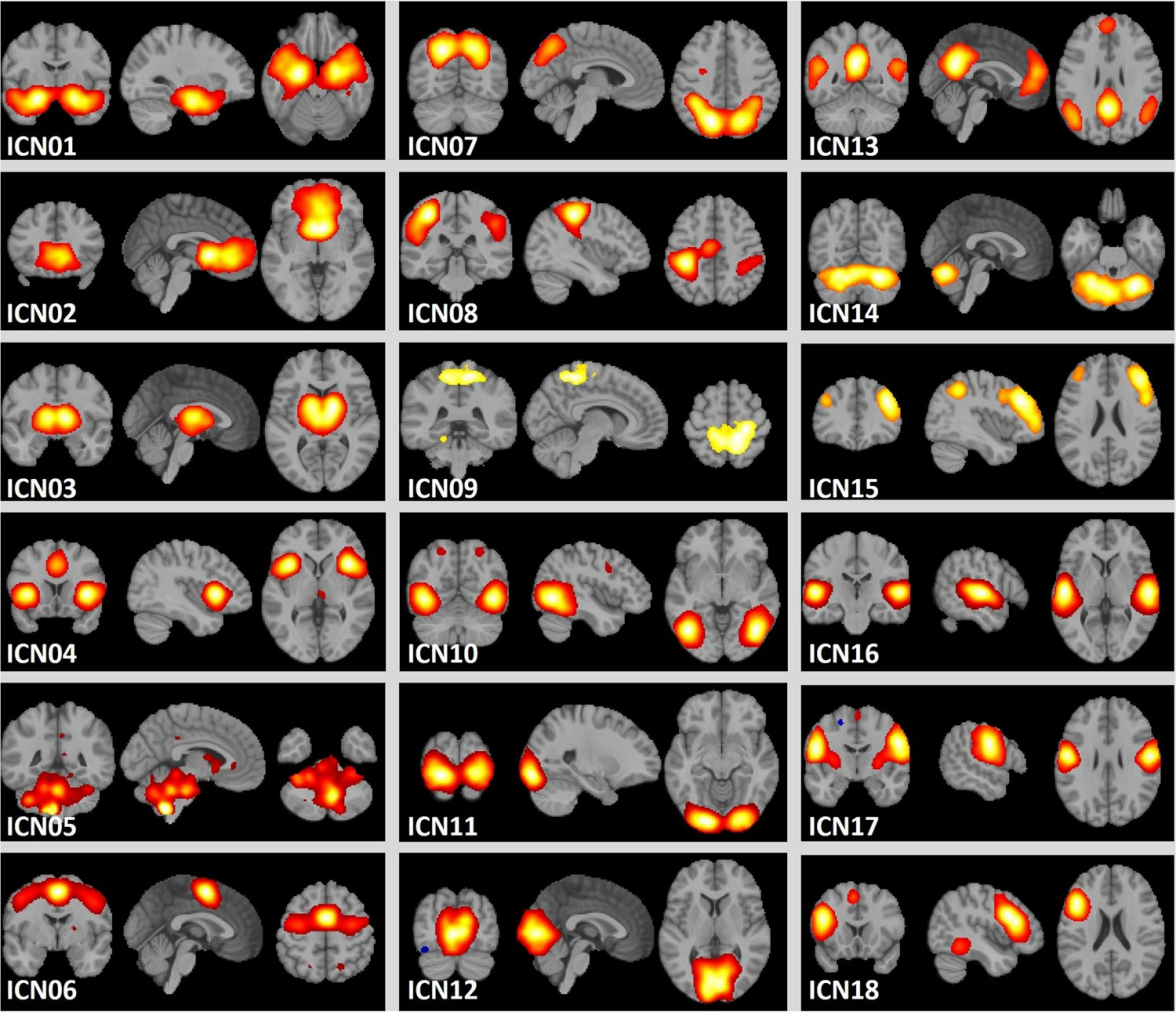
Visualization of the ICNs. The 18 ICNs based on Laird et al. [12] are illustrated. ICNs are *z*-standardized, thresholded at *z* > 4 and mapped onto the MNI152NLin2009cAsym standard space with a resolution of 2 mm in neurological convention. Color mapping is not standardized across ICNs

Due to differences in various scanning parameters, all analyses were executed separately for data from Munich and Mannheim. Table 2 and Fig. 2 illustrate the 18 ICNs with their included regions proposed by Laird et al. [12].

**Table 2:** ICNs and the corresponding anatomical regions

Group	Network	Regions
1	ICN01	Limbic and medial-temporal areas
Emotional and autonomic processes	ICN02	Subgenual anterior cingulate cortex, orbitofrontal cortex
	ICN03	Basal ganglia and thalamus
	ICN04	Anterior insula, frontal opercula, anterior cingulate cortex (SN)
	ICN05	Midbrain
2	ICN06	Premotor and supplementary motor area, frontal eye field
Motor and visuospatial integration, coordination, execution	ICN07	Dorsolateral prefrontal cortex, posterior parietal cortex
	ICN08	Primary sensorimotor cortices (extremities)
	ICN09	Medial posterior parietal association areas
3	ICN10	Middle temporal visual association areas
Visual perception	ICN11	Primary, secondary and tertiary visual cortices
	ICN12	Primary, secondary and tertiary visual cortices
4	ICN13	Medial prefrontal and posterior cingulate cortex (DMN)
Divergent networks	ICN14	Cerebellum
	ICN15	Right-lateralized fronto-parietal regions (right FPN)
	ICN16	Transverse temporal gyri, primary auditory cortices
	ICN17	Primary sensorimotor cortices (mouth)
	ICN18	Left-lateralized fronto-parietal regions (left FPN)

The 18 ICNs based on Laird et al. [12] including the anatomical regions that they cover are listed

#### Between-ICN connectivity

Using *melodic* from FSL v6.0.4 [44, 48], a group ICA was computed to extract 20 sample-specific independent components (ICs). *Dual regression* from FSL v6.0.4 [44, 48] was administered to extract the BOLD-timeseries of every IC for each subject. *ConnectivityMeasure* function from Nilearn v0.8.0 was used to calculate subject-specific FC between ICs quantified as Pearson’s correlation coefficient. The latter were converted to *z* values using Fisher’s *r*-to-*z* transformation. ICs were cross-correlated with the 18 ICNs of the functional atlas provided by Laird et al. [12] utilizing *fslcc* of FSL v6.0.4 [44]. Each IC was assigned to a corresponding ICN depending on the magnitude of their statistical overlap (*r* ≥ 0.2). In ambiguous cases, ICs were inspected visually and assignments were adjusted if necessary (see suppl. S4 for cross-correlation results and ICN/IC visualizations). Data from study sites Munich and Mannheim were concatenated after ICN-assignments. In sum, 148 between-ICN FC measures per subject (one for every unique ICN combination) were computed.

#### Within-ICN connectivity

The *melodic_IC* output from the abovementioned group ICA was splitted using *fslsplit* from FSL v6.0.4 [44]. The created single IC files were thresholded (*z* = 4) and binarized with *fslmaths* providing masks of every IC. Canonical ICA [49] within those masks was performed with the *CanICA* function of Nilearn v0.8.0 aiming at extracting two sub-ICs within every main IC. To determine the BOLD-timeseries of each sub-IC at single-subject level, the *fit_transform* attribute from Nilearn v0.8.0 was applied. FC within every IC was again calculated with the *ConnectivityMeasure* function from Nilearn v0.8.0 by correlating the timeseries of both corresponding sub-ICs and performing Fisher’s transformation. Data from study sites Munich and Mannheim were merged and site-specific ICs were labelled based on ICN-assignments. In total, 18 within-ICN FC measures per subject (one for each ICN) were extracted.

#### Seed-based connectivity

*NiftiLabelsMasker* function from Nilearn v0.8.0 was employed to extract the BOLD-timeseries of 116 brain regions defined by the Automated Anatomical Labelling (AAL) atlas [50]. FC between these regional, subject-specific timeseries was again calculated using *ConnectivityMeasure* function from Nilearn v0.8.0 and Fisher’s transformation. Data from both study centers were concatenated analogously. In sum, 6670 seed-based FC measures per subject (one for every unique AAL-region combination) were calculated.

### Clinical and cognitive data acquisition

The Positive and Negative Syndrome Scale (PANSS) [51] was employed to assess positive (PANSS-positive), negative (PANSS-negative) and general psychopathological symptoms (PANSS-psychopath) as well as summarized symptom severity (PANSS-total). Calgary Depression Scale for Schizophrenia (CDSS) was utilized to measure depressive symptoms [52]. Covering global disorder severity, the Clinical Global Impression (CGI) scale was administered [53]. Global cognition was targeted by Trail Making Tests A and B (TMT) [54], the category naming part of the Brief Cognitive Assessment Tool for Schizophrenia (B-CATS) [55] and the Digit Symbol Substitution Test (DSST) [56]. The forward and backward versions of the Digit Span Test (DST) [56] were used to measure verbal working memory performance, while verbal declarative memory was covered by seven different measures of the Verbal Learning and Memory Test (VLMT) [57]. Emotion recognition capability was examined by an adjusted version of the Emotion Recognition Test (ERT) [58]. For detailed descriptions of the co gnitive tests and the corresponding abbreviations see suppl. S5.

### Statistical data analysis

Rstudio v1.4.1717 based on R v4.1.2 was used for statistical data analysis [59, 60]. We detected outliers in the distributions of FC data as well as clinical and cognitive data (for details see suppl. S6). Thereafter, behavioral data were *z*-standardized and multilevel Bayesian partial correlations between aerobic fitness and all functional connections (between-ICN: 148 connections, within-ICN: 18, seed-based: 6670) were calculated using the *correlation* package in R v4.1.2 [61]. Age, body-mass-index (BMI), disorder duration, education years and chlorpromazine equivalents were included as covariates, while sex and study site were treated as random factors within a mixed effect model. Chlorpromazine equivalents were computed based on the defined daily dose method [62]. The main output of interest was Jeffrey’s default Bayes factor (BF_10_) representing a continuous, relative measure of evidence the data is providing for the alternative hypothesis (H_1_: *r* ≠ 0) compared to the null hypothesis (H_0_: *r* = 0) [63, 64]. For instance, if the BF_10_ = 3, it is three times more likely to observe the current data under the alternative hypothesis than under the null hypothesis. The BF_10_ can be separated in different categories of evidence strength facilitating interpretations and conclusions (suppl. tab. S7) [65]. In addition, Pearson’s correlation coefficient with its corresponding highest density interval (HDI), the probability of direction (PD) and the region of practical equivalence (ROPE) were considered to evaluate the existence of an association between the variables of interest [66] (for detailed description of Bayesian parameters and prior selection see suppl. S7). Regarding between- and within-ICN FC, we focused on single associations between aerobic fitness and the corresponding functional connection. We correlated between- and within-ICN connections with clinical and cognitive scores, if they tended to relate to aerobic fitness. Considering the large number of 6670 FC measures in the seed-based approach, we examined if seed connections of specific anatomical clusters defined by the AAL atlas [50] were related to aerobic fitness most robustly (for a detailed description of cluster definition and evaluation of robustness see suppl. S8). The most prominent anatomical clusters were related to clinical and cognitive scores. In all three approaches, the *mediation* package in R v4.1.2 [67] was used to compute a mediation analysis, if correlations between aerobic fitness and FC as well as FC and clinical/cognitive scores existed. Finally, a Bayes factor design analysis (BFDA) [68, 69] was performed aiming to evaluate the probabilities to obtain a BF_10_ > 3 under the alternative and null hypothesis within the current study design. BFDA was done utilizing the *BFDA* package in R v4.1.2 [70] (see suppl. S9).

## Results

### Aerobic fitness, between-ICN connectivity and clinical/cognitive outcome

**Fig. 3:**
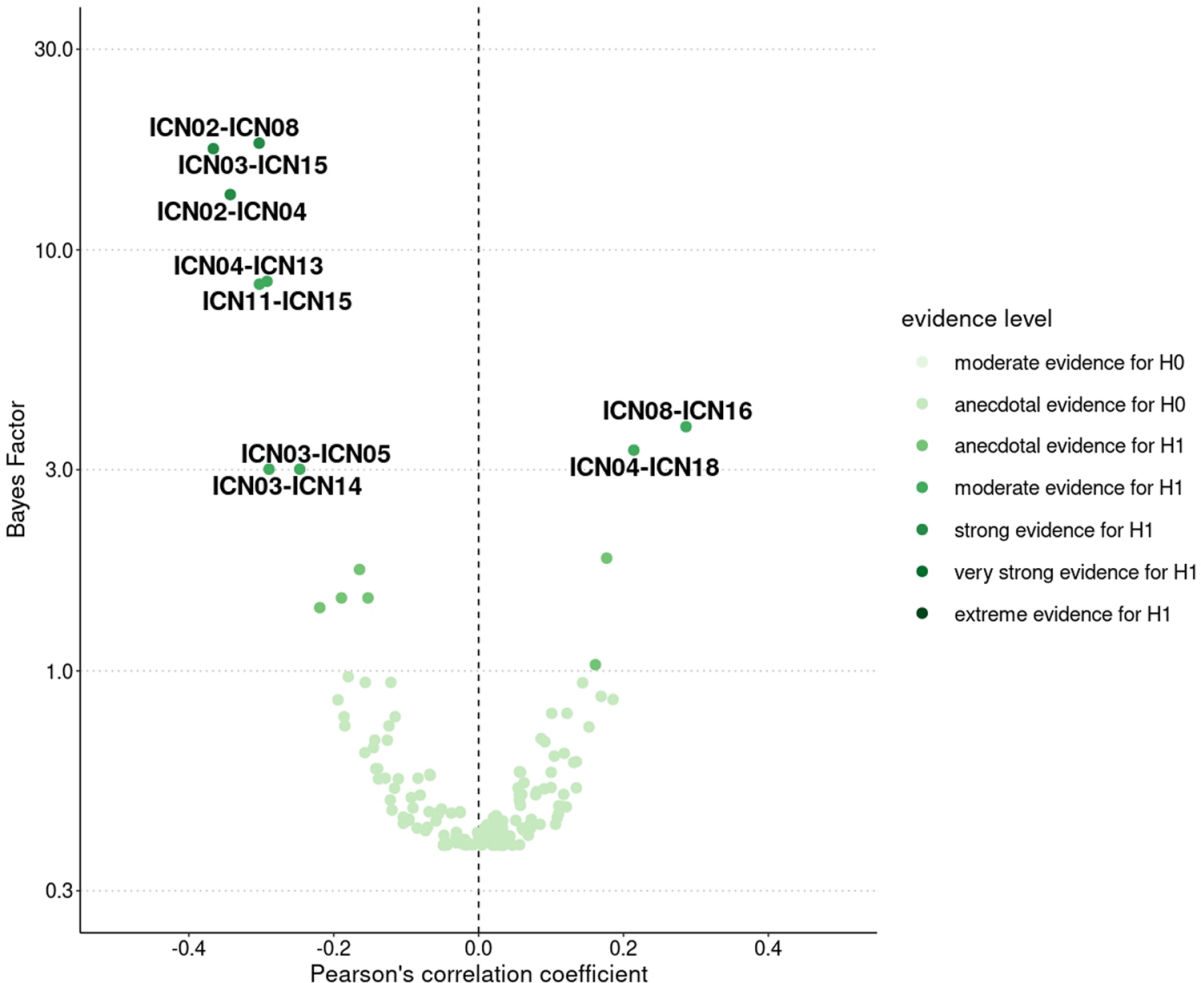
BFs and partial correlations between aerobic fitness and between-ICN connectivity. Correlation tests resulting in a BF_10_ around three or higher are labelled with the corresponding between-ICN connection. Categorical scheme of the BF_10_ according to Lee and Wagenmarkers [65]: BF_10_ > 1: anecdotal evidence for H_1_, BF_10_ > 3: moderate evidence for H_1_, BF_10_ > 10: strong evidence for H_1_, BF_10_ > 30: very strong evidence for H_1_, BF_10_ > 100: extreme evidence for H_1_, *N* = 58

15 of 148 investigated functional connections demonstrated at least anecdotal evidence in favor of a correlation to aerobic fitness (BF_10_ > 1, Fig. 3). The following eight functional connections revealed the most robust associations ranging from moderate to very strong evidence strengths. ICN05 and ICN14 were covered by the same IC in our sample (suppl. S4) resulting in equal findings:

Aerobic fitness was positively correlated with FC between ICN08 (primary sensorimotor cortices) and ICN16 (primary auditory cortices) (BF_10_ = 3.80, *r* = 0.26 [0.06, 0.43], PD = 98.6%, ROPE = 9.4%) as well as ICN04 (SN) and ICN18 (left FPN) (BF_10_ = 3.34, *r* = 0.25 [0.05, 0.42], PD = 98.3%, ROPE = 10.4%). Aerobic fitness revealed negative correlations with FC between ICN02 (subgenual anterior cingulate and orbitofrontal cortex) and ICN08 (primary sensorimotor cortices) (BF_10_ = 17.94, *r* = − 0.33 [− 0.50, − 0.17], PD = 99.9%, ROPE = 2.3%), ICN03 (basal ganglia and thalamus) and ICN15 (right FPN) (BF_10_ = 17.41, *r* = − 0.33 [− 0.50, − 0.17], PD = 99.9%, ROPE = 2.4%), ICN02 (subgenual anterior cingulate and orbitofrontal cortex) and ICN04 (SN) (BF_10_ = 3.09, *r* = − 0.25 [− 0.44, − 0.07], PD = 98.0%, ROPE = 11.3%), ICN04 (basal ganglia and thalamus) and ICN13 (DMN) (BF_10_ = 13.54, *r* = − 0.32 [− 0.49, − 0.15], PD = 99.8%, ROPE = 2.6%), ICN11 (visual cortices) and ICN15 (right FPN) (BF_10_ = 8.29, *r* = − 0.30 [− 0.47, − 0.12], PD = 99.2%, ROPE = 4.2%) and ICN03 (basal ganglia and thalamus) and ICN05/14 (midbrain and cerebellum) (BF_10_ = 3.01, *r* = − 0.24 [− 0.43, − 0.07], PD = 97.9%, ROPE = 11.7%).

**Fig. 4:**
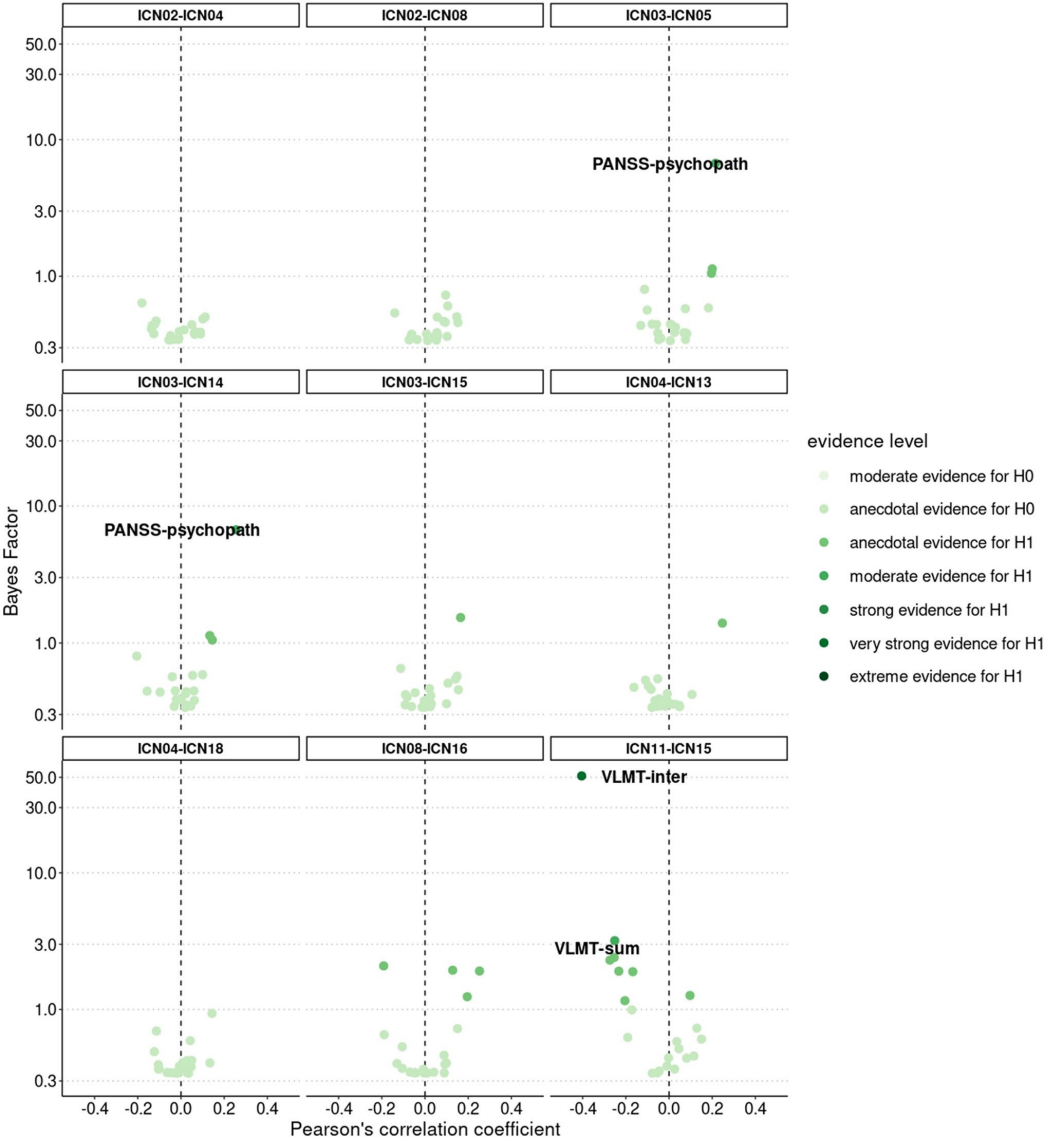
BFs and partial correlations between-ICN FC and clinical and cognitive scores. Correlation tests resulting in a BF_10_ around three or higher are labelled with the corresponding name of the test battery. Categorical scheme of the BF_10_ according to Lee and Wagenmarkers [65]: BF_10_ > 1: anecdotal evidence for H_1_, BF_10_ > 3: moderate evidence for H_1_, BF_10_ > 10: strong evidence for H_1_, BF_10_ > 30: very strong evidence for H_1_, BF_10_ > 100: extreme evidence for H_1_, *N* = 72–79

Two of the eight functional connections linked to aerobic fitness were associated with clinical or cognitive scores (Fig. 4) accompanied by two significant mediation effects:

The functional connection between ICN03 (basal ganglia and thalamus) and ICN05/14 (midbrain and cerebellum) was positively correlated with PANSS-psychopath (BF_10_ = 6.71, *r* = 0.25 [0.08, 0.40], PD = 99.1%, ROPE = 7.2%) including a significant negative mediation effect (*p* = 0.022, *β* = − 0.15 [− 0.30, − 0.03]). Hence, higher patients’ aerobic fitness levels were accompanied by lower FC between the basal ganglia/thalamus and the midbrain/cerebellum, which in turn led to lower PANSS-psychopath scores. The functional connection between ICN11 (visual cortices) and ICN15 (right FPN) revealed a negative correlation with VLMT-inter (BF_10_ = 51.15, *r* = − 0.34 [− 0.49, − 0.19], PD = 100%, ROPE = 1.2%) and VLMT-sum score (BF_10_ = 3.19, *r* = − 0.23 [− 0.39, − 0.07], PD = 98.4%, ROPE = 12.1%). Positive mediation effects were significant in case of VLMT-inter (*p* = 0.044, *β* = 0.13 [0.18, 0.27]), but not for VLMT-sum (*p* = 0.25, *β* = 0.05 [− 0.01, 0.13]). Consequently, the higher patients’ aerobic fitness was, the lower was FC between the visual network and the right FPN leading to better performance in VLMT-inter.

### Aerobic fitness, within-ICN connectivity and clinical/cognitive outcome

**Fig. 5:**
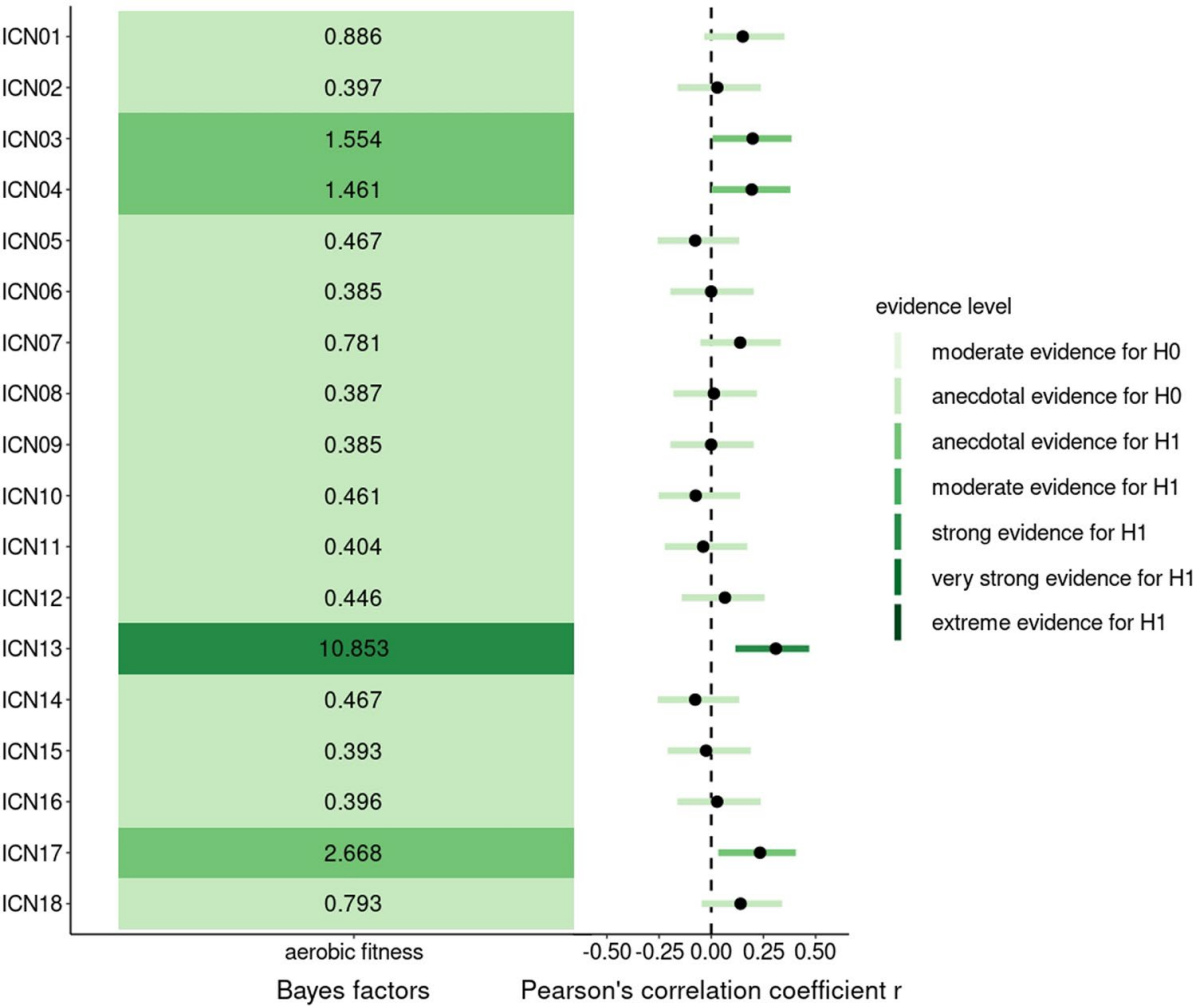
BFs and partial correlations between aerobic fitness and within-ICN connectivity. On the left-hand side, BFs of the multilevel partial correlation tests between aerobic fitness and within-ICN FC are displayed and colored according to evidence strength. On the right, the corresponding correlation coefficients and the HDIs are visualized. Categorical scheme of the BF_10_ according to Lee and Wagenmarkers [65]: BF_10_ > 1: anecdotal evidence for H_1_, BF_10_ > 3: moderate evidence for H_1_, BF_10_ > 10: strong evidence for H_1_, BF_10_ > 30: very strong evidence for H_1_, BF_10_ > 100: extreme evidence for H_1_, *N* = 58

Four of 18 investigated functional connections indicated at least anecdotal evidence in favor of a correlation to aerobic fitness (BF_10_ > 1, Fig. 5). The most robust finding was the positive correlation between aerobic fitness and FC within ICN13 (DMN) (BF_10_ = 10.85, *r* = 0.31 [0.12, 0.47], PD = 99.4%, ROPE = 3.7%) (Fig. 5).

**Fig. 6:**
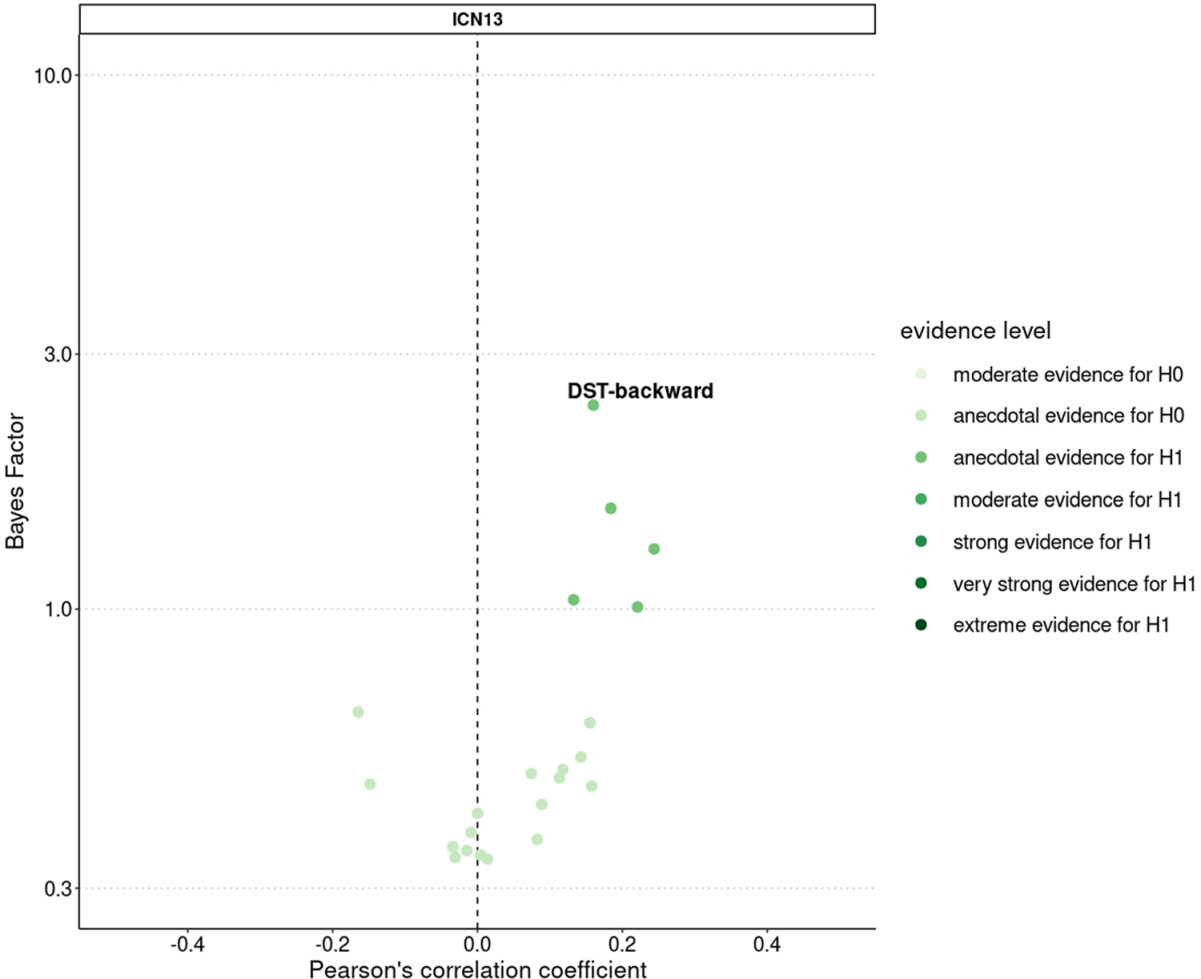
BFs and partial correlations between within-ICN FC and clinical and cognitive scores. Visualization of the BFs and correlation coefficients of the Bayesian multilevel partial correlation tests including within-ICN connectivity and clinical and cognitive scores. Correlation tests resulting in a BF_10_ of two or higher are labelled with the corresponding name of the test battery. Categorical scheme of the BF_10_ according to Lee and Wagenmarkers [65]: BF_10_ > 1: anecdotal evidence for H_1_, BF_10_ > 3: moderate evidence for H_1_, BF_10_ > 10: strong evidence for H_1_, BF_10_ > 30: very strong evidence for H_1_, BF_10_ > 100: extreme evidence for H_1_, *N* = 72–79

With respect to clinical and cognitive relevance (Fig. 6), anecdotal evidence in favour of a positive correlation between FC within ICN13 (DMN) and DST-backward performance (BF_10_ = 2.41, *r* = 0.21 [0.04, 0.38], PD = 97.7%, ROPE = 14.7%) was observed, but a significant mediation effect was lacking (*p* = 0.21, *β* = 0.07 [− 0.01, 0.17]).

### Aerobic fitness, seed-based connectivity and clinical/cognitive outcome

**Fig. 7:**
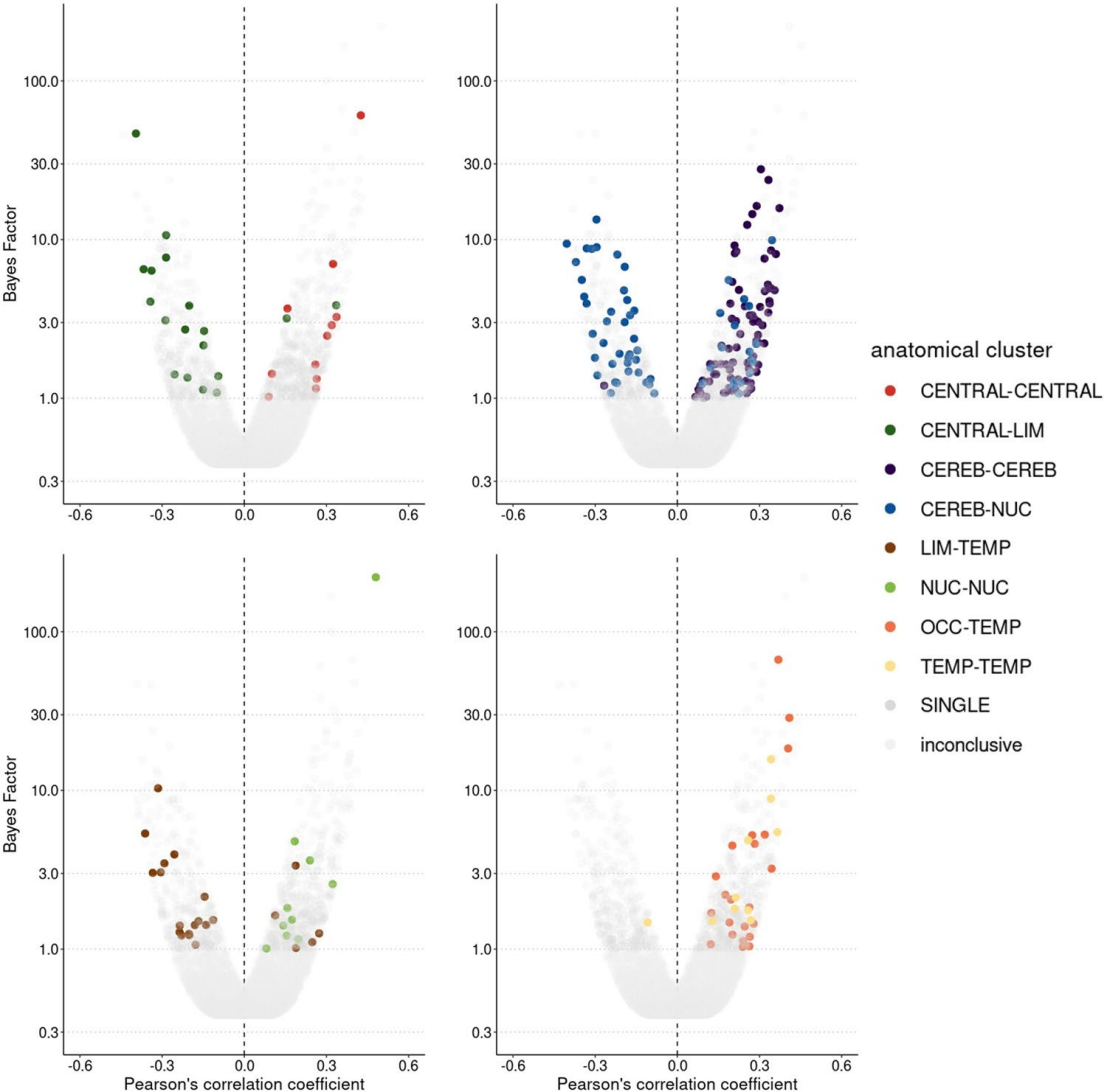
BFs and partial correlations between aerobic fitness and seed-based connectivity of anatomical clusters. Tests resulting in a BF_10_ < 1 are labelled as inconclusive. Tests resulting in a BF_10_ > 1 are either assigned to the eight most consistent clusters CENTRAL–CENTRAL, CENTRAL–LIM, CEREB–CEREB, CEREB–NUC, LIM–TEMP, NUC–NUC, OCC–TEMP, TEMP–TEMP or labelled as SINGLE. *N* = 58, CENTRAL: central cortical structures, LIM: limbic lobe, CEREB: cerebellum, NUC: subcortical nuclei, TEMP: temporal lobe, OCC: occipital lobe, SINGLE = single connections that were linked to aerobic fitness, but were not part of a robust cluster

The 6670 functional connections between all AAL-regions were assigned to 45 anatomical clusters based on the anatomical description proposed by Tzourio-Mazoyer et al. [50]. These 45 anatomical clusters were evaluated in terms of the robustness of their relation to aerobic fitness (suppl. S8). Eight anatomical clusters demonstrated the most robust associations with aerobic fitness (Fig. 7):

The first cluster consisted of 15 functional connections between seeds from central cortical regions (CENTRAL–CENTRAL). Eleven of these 15 functional connections (73.3%) were linked to aerobic fitness. All eleven correlations were positive (100%) and evidence strengths ranged from anecdotal to very strong (BF_10mean_ = 7.87, BF_10median_ = 2.48, BF_10range_ = 1.02–60.66, *r* = 0.16–0.38, PD = 91.5–100%, ROPE = 27.5–1.2%).

The second cluster comprised 84 functional connections between seeds from central cortical regions and seeds from the limbic lobe (CENTRAL–LIM). 18 of these 84 functional connections (21.43%) revealed correlations with aerobic fitness. 16 correlations were negative (88.89%) accompanied by anecdotal to very strong evidence strengths (BF_10mean_ = 6.42, BF_10median_ = 2.91, BF_10range_ = 1.09–46.60, *r* = − 0.17 to − 0.37, PD = 92.3–100%, ROPE = 25.0–1.0%).

325 functional connections between cerebellar seeds formed the third cluster (CEREB–CEREB). 80 of these 325 functional connections (24.62%) demonstrated correlations with aerobic fitness. 79 correlations were positive (98.75%) ranging from anecdotal to strong evidence levels (BF_10mean_ = 3.94, BF_10median_ = 2.09, BF_10range_ = 1.00–27.76, *r* = 0.16–0.35, PD = 91.3–99.8%, ROPE = 28.1–1.8%).

The fourth cluster included 260 functional connections between cerebellar seeds and seeds from the subcortical nuclei (CEREB–NUC). 57 of these 260 functional connections (21.92%) revealed correlations with aerobic fitness. 39 correlations were negative (68.42%) including anecdotal to strong evidence strengths (BF_10mean_ = 3.72, BF_10median_ = 2.37, BF_10range_ = 1.07–13.34, *r* = − 0.17 to − 0.32, PD = 92.2–99.8%, ROPE = 25.4–2.7%).

The fifth cluster consisted of 112 functional connections between seeds from the limbic lobe and seeds from the temporal lobe (LIM-TEMP). 22 of these 112 functional connections (19.64%) correlated with aerobic fitness. 17 correlations were negative (77.27%) and evidence levels ranged from anecdotal to strong (BF_10mean_ = 2.62, BF_10median_ = 1.50, BF_10range_ = 1.07–10.32, *r* = − 0.17 to − 0.31, PD = 92.2–99.5%, ROPE = 25.6–3.5%).

45 functional connections between seeds from the subcortical nuclei formed the sixth cluster (NUC–NUC). Ten of these 45 functional connections (22.22%) exhibited correlations with aerobic fitness. All ten correlations were positive (100%) ranging from anecdotal to extreme evidence levels (BF_10mean_ = 23.96, BF_10median_ = 1.67, BF_10range_ = 1.01–220.48, *r* = 0.16–0.42, PD = 91.3–100%, ROPE = 28.0–0.3%).

The seventh cluster comprised 112 functional connections between seeds from the occipital lobe and seeds from the temporal lobe (OCC–TEMP). 22 of these 112 functional connections (19.64%) revealed correlations with aerobic fitness. All 22 correlations were positive (100%) accompanied by anecdotal to very strong evidence strengths (BF_10mean_ = 7.19, BF_10median_ = 1.94, BF_10range_ = 1.04–66.69, *r* = 0.16 0.38, PD = 91.7–100%, ROPE = 26.9–1.4%).

The eighth cluster included 28 functional connections between seeds from the temporal lobe (TEMP–TEMP). Ten of these 28 functional connections (35.71%) correlated with aerobic fitness. Nine of ten correlations were positive (90%) ranging from anecdotal to strong evidence levels (BF_10mean_ = 4.85, BF_10median_ = 2.11, BF_10range_ = 1.50–15.71, *r* = 0.20–0.33, PD = 95.3–99.5%, ROPE = 19.4–2.8%).

Of these eight anatomical clusters linked to aerobic fitness, the following five exhibited consistent relations to clinical and cognitive outcomes across multiple functional connections (suppl. figs. S10.2, S10.3, S10.4, S10.7 and S10.8):

Four of 18 functional connections linked to aerobic fitness in the CENTRAL-LIM cluster correlated with performance in the ERT (BF_10range_ = 6.93–41.53, *r* = − 0.26 to − 0.33, PD = 99.1–100%, ROPE = 6.0–1.4%). Two of four mediation effects were significant suggesting a positive impact of aerobic fitness on ERT performance mediated by two functional connections between seeds from central cortical regions and seeds from limbic lobe (Table 3).

**Table 3:** Results of the mediation analyses

IV	Mediator	DV	Eff	Estimate	CI low	CI high	*p*	*n*
CENTRAL–LIM								
Aerobic fitness	CingulumAntR-PostcentralR	ERT	ACME	0.03	− 0.04	0.12	0.550	55
Aerobic fitness	RolandicOperL-CingulumPostR	ERT	ACME	0.09	0.004	0.21	0.080	55
Aerobic fitness	RolandicOperR-CingulumPostL	ERT	ACME	0.15	0.03	0.30	0.040*	55
Aerobic fitness	RolandicOperR-CingulumPostR	ERT	ACME	0.12	0.02	0.25	0.036*	55
CEREB–CEREB								
Aerobic fitness	Cerebellum3R-Cerebellum45L	TMT-A	ACME	− 0.02	− 0.10	0.06	0.740	57
Aerobic fitness	Cerebellum9R-Vermis10	TMT-A	ACME	− 0.10	− 0.21	− 0.02	0.028*	57
Aerobic fitness	CerebellumCrus1R-Cerebellum6L	TMT-A	ACME	− 0.05	− 0.14	0.00	0.130	57
Aerobic fitness	CerebellumCrus2R-Cerebellum10R	TMT-A	ACME	− 0.04	− 0.11	0.01	0.31	57
Aerobic fitness	CerebellumCrus2R-Cerebellum9L	TMT-A	ACME	− 0.11	− 0.23	− 0.03	0.024*	57
Aerobic fitness	CerebellumCrus2R-Cerebellum9L	TMT-B	ACME	− 0.12	− 0.24	− 0.02	0.044*	56
Aerobic fitness	CerebellumCrus2R-Vermis9	TMT-A	ACME	− 0.06	− 0.15	0.00	0.098	57
Aerobic fitness	Cerebellum3R-Cerebellum45R	B-CATS-fruits	ACME	0.05	− 0.02	0.14	0.320	55
Aerobic fitness	Cerebellum7bR-Cerebellum9L	B-CATS-fruits	ACME	0.08	0.00	0.21	0.11	55
Aerobic fitness	Cerebellum7bR-Cerebellum9L	B-CATS-vegetables	ACME	0.06	− 0.03	0.19	0.290	51
Aerobic fitness	Cerebellum7bR-Vermis9	B-CATS-fruits	ACME	0.05	− 0.02	0.15	0.280	55
Aerobic fitness	Cerebellum7bR-Vermis9	B-CATS-vegetables	ACME	0.03	− 0.03	0.11	0.550	51
Aerobic fitness	Cerebellum9L-Vermis7	B-CATS-vegetables	ACME	0.10	− 0.01	0.23	0.146	51
Aerobic fitness	Cerebellum9L-Vermis7	B-CATS-animals	ACME	0.06	− 0.01	0.16	0.244	55
Aerobic fitness	Cerebellum9L-Vermis8	B-CATS-vegetables	ACME	0.10	0.00	0.22	0.098	51
Aerobic fitness	Cerebellum9L-Vermis8	B-CATS-animals	ACME	0.08	− 0.01	0.19	0.140	55
Aerobic fitness	Cerebellum9R-Vermis7	B-CATS-vegetables	ACME	0.12	0.01	0.27	0.054	51
Aerobic fitness	Cerebellum9R-Vermis8	B-CATS-vegetables	ACME	0.13	0.02	0.28	0.050	51
Aerobic fitness	Cerebellum9R-Vermis9	B-CATS-animals	ACME	0.09	− 0.01	0.21	0.166	55
Aerobic fitness	CerebellumCrus1R-Vermis6	B-CATS-animals	ACME	0.00	− 0.11	0.12	0.980	55
Aerobic fitness	CerebellumCrus2R-Cerebellum9L	B-CATS-vegetables	ACME	0.04	− 0.04	0.15	0.450	51
CEREB–NUC								
Aerobic fitness	AmygdalaR-CerebellumCrus2L	PANSS-negative	ACME	− 0.07	− 0.18	0.00	0.140	57
Aerobic fitness	CaudateL-CerebellumCrus1R	PANSS-negative	ACME	− 0.09	− 0.21	0.00	0.140	57
Aerobic fitness	CaudateR-Cerebellum45L	PANSS-positive	ACME	− 0.07	− 0.19	0.00	0.120	58
Aerobic fitness	CaudateR-Cerebellum45L	PANSS-total	ACME	− 0.06	− 0.16	0.01	0.220	58
Aerobic fitness	CaudateR-Cerebellum45L	CDSS	ACME	− 0.09	− 0.20	− 0.01	0.044*	58
Aerobic fitness	PallidumR-Vermis3	CDSS	ACME	0.03	− 0.3	0.11	0.420	58
Aerobic fitness	PallidumR-Vermis8	CGI	ACME	− 0.09	− 0.21	0.00	0.110	58
Aerobic fitness	PallidumR-Vermis8	PANSS-total	ACME	− 0.08	− 0.21	0.00	0.120	58
Aerobic fitness	PallidumR-Vermis8	PANSS-psychopath	ACME	− 0.08	− 0.18	0.00	0.140	58
Aerobic fitness	PallidumR-Vermis8	PANSS-positive	ACME	− 0.05	− 0.15	0.01	0.250	58
Aerobic fitness	PutamenL-Vermis45	PANSS-psychopath	ACME	0.05	− 0.01	0.15	0.250	58
Aerobic fitness	PutamenL-Vermis45	CDSS	ACME	0.05	− 0.01	0.13	0.230	58
Aerobic fitness	ThalamusL-Cerebellum9R	CGI	ACME	− 0.06	− 0.16	0.01	0.25	58
Aerobic fitness	ThalamusR-Cerebellum9R	CGI	ACME	− 0.15	− 0.30	− 0.04	0.022*	58
Aerobic fitness	CaudateL-Cerebellum6L	B-CATS-fruits	ACME	0.05	− 0.01	0.15	0.260	55
Aerobic fitness	CaudateL-Cerebellum6L	B-CATS-vegetables	ACME	0.02	− 0.05	0.11	0.574	51
Aerobic fitness	CaudateL-Cerebellum6R	B-CATS-vegetables	ACME	0.05	− 0.06	0.18	0.500	51
Aerobic fitness	CaudateR-Cerebellum8L	B-CATS-fruits	ACME	0.04	− 0.02	0.14	0.330	55
Aerobic fitness	PallidumR-CerebellumCrus1L	B-CATS-fruits	ACME	0.08	− 0.02	0.20	0.220	55
Aerobic fitness	PutamenL-CerebellumCrus1L	B-CATS-fruits	ACME	0.07	− 0.02	0.20	0.230	55
Aerobic fitness	PutamenL-CerebellumCrus2L	B-CATS-fruits	ACME	0.03	− 0.05	0.12	0.610	55
Aerobic fitness	PutamenR-Cerebellum10L	B-CATS-vegetables	ACME	0.05	− 0.02	0.15	0.316	51
Aerobic fitness	PutamenR-CerebellumCrus1L	B-CATS-fruits	ACME	0.07	− 0.01	0.17	0.190	55
Aerobic fitness	CaudateL-Cerebellum7bL	DSST	ACME	− 0.14	− 0.27	− 0.03	0.018*	56
Aerobic fitness	CaudateL-CerebellumCrus1L	DSST	ACME	− 0.09	− 0.19	− 0.01	0.076	56
Aerobic fitness	CaudateL-CerebellumCrus1R	DSST	ACME	− 0.14	− 0.28	− 0.04	0.022*	56
Aerobic fitness	CaudateR-CerebellumCrus1L	DSST	ACME	− 0.09	− 0.20	− 0.01	0.074	56
Aerobic fitness	PallidumL-Cerebellum6L	DSST	ACME	0.03	− 0.02	0.10	0.390	56
Aerobic fitness	PallidumL-Cerebellum8L	DSST	ACME	0.08	0.00	0.18	0.090	56
Aerobic fitness	PallidumL-Cerebellum8R	DSST	ACME	0.09	0.01	0.20	0.060	56
Aerobic fitness	PallidumL-Vermis6	DSST	ACME	0.03	− 0.02	0.10	0.440	56
Aerobic fitness	PallidumL-Vermis7	DSST	ACME	0.09	0.00	0.20	0.100	56
Aerobic fitness	ThalamusR-Cerebellum9R	DSST	ACME	0.14	0.04	0.27	0.008*	56
OCC–TEMP								
Aerobic fitness	OccipitalInfL-HeschlL	PANSS-negative	ACME	0.08	0.00	0.21	0.138	57
Aerobic fitness	OccipitalInfL-HeschlL	PANSS-total	ACME	0.11	0.01	0.25	0.054	58
Aerobic fitness	OccipitalMidL-HeschlL	PANSS-psychopath	ACME	0.09	0.00	0.20	0.110	58
Aerobic fitness	OccipitalMidL-HeschlR	PANSS-psychopath	ACME	0.07	0.00	0.18	0.140	58
Aerobic fitness	OccipitalMidL-HeschlR	PANSS-total	ACME	0.06	− 0.01	0.17	0.190	58
Aerobic fitness	OccipitalMidR-TemporalMidL	PANSS-negative	ACME	0.10	0.00	0.22	0.088	57
Aerobic fitness	OccipitalSupL-HeschlL	PANSS-psychopath	ACME	0.07	0.00	0.18	0.150	58
Aerobic fitness	OccipitalSupL-HeschlR	PANSS-psychopath	ACME	0.09	0.00	0.20	0.110	58
Aerobic fitness	OccipitalSupL-HeschlR	CGI	ACME	0.09	0.00	0.20	0.100	58
Aerobic fitness	OccipitalSupL-HeschlR	PANSS-total	ACME	0.07	0.00	0.19	0.130	58
Aerobic fitness	OccipitalSupR-HeschlR	PANSS-psychopath	ACME	0.07	− 0.01	0.17	0.180	58
Aerobic fitness	OccipitalSupR-HeschlR	CGI	ACME	0.07	0.00	0.18	0.150	58
Aerobic fitness	OccipitalSupR-TemporalMidL	PANSS-negative	ACME	0.09	0.00	0.21	0.122	57
TEMP–TEMP								
Aerobic fitness	HeschlR-TemporalMidL	PANSS-total	ACME	− 0.14	− 0.29	− 0.02	0.024*	58
Aerobic fitness	HeschlR-TemporalMidL	CGI	ACME	− 0.14	− 0.29	− 0.03	0.024*	58
Aerobic fitness	HeschlR-TemporalMidL	PANSS-positive	ACME	− 0.13	− 0.27	− 0.03	0.022*	58
Aerobic fitness	TemporalSupR-TemporalMidR	PANSS-negative	ACME	− 0.14	− 0.28	− 0.02	0.044*	58

Summary of the results of the mediation analyses. IV = independent variable, DV = dependent variable, Eff. = type of the effect, estimate = β-coefficient of the mediation effect, CI = confidence interval, n = sample size, ACME = average causal mediation effect, CENTRAL–LIM = functional connections between seeds from central cortical structures and limbic lobe, CEREB–CEREB = functional connections between cerebellar seeds, CEREB–NUC = functional connections between cerebellar seeds and seeds from the subcortical nuclei, OCC–TEMP = functional connections between seeds from the occipital lobe and temporal lobe, TEMP–TEMP = functional connections between seeds from the temporal lobe

*p < 0.05

Six of 80 functional connections related to aerobic fitness in the CEREB–CEREB cluster correlated with performance in the TMT-A and -B (BF_10range_ = 2.89–82.52, *r* = − 0.22 to − 0.35, PD = 98.3–100%, ROPE = 12.6–0.9%). Three of seven mediation effects were significant proposing a positive impact of aerobic fitness on TMT speed mediated by three functional connections between cerebellar seeds (Table 3). Further nine functional connections in the CEREB-CEREB cluster correlated with B-CATS-animals, -fruits and -vegetables (BF_10range_ = 2.53–15.26, *r* = 0.21–0.30, PD = 97.7–99.5%, ROPE = 13.8–3.0%). No significant mediation effects were observed (Table 3).

Five of 57 functional connections related to aerobic fitness in the CEREB–NUC cluster correlated with the four PANSS scores as well as CDSS and CGI score (BF_10range_ = 2.52–16.36, *r* = − 0.28 to 0.29, PD = 98.1–99.6%, ROPE = 13.9–2.9%). Two of 14 mediation effects were significant underlying an attenuating effect of aerobic fitness on total symptom severity mediated by two functional connections between cerebellar seeds and seeds from subcortical nuclei (Table 3). Further eight functional connections in the CEREB-NUC cluster correlated with B-CATS-fruits and -vegetables (BF_10range_ = 3.60–24.88, *r* = − 0.23 to − 0.31, PD = 98.3–100%, ROPE = 11.1–2.0%). No significant mediation effects were found (Table 3). Finally, ten functional connections in the CEREB-NUC cluster showed correlations with DSST performance (BF_10range_ = 2.94–35.09, *r* = 0.22–0.32, PD = 98.1–99.9%, ROPE = 12.7–1.4%). Three of ten mediation effects were significant suggesting both a positive and a negative influence of aerobic fitness on DSST performance mediated by three functional connections between cerebellar seeds and seeds from the subcortical nuclei (Table 3).

Eight of 22 functional connections associated with aerobic fitness in the OCC-TEMP cluster correlated with three PANSS scores as well as CGI score (BF_10range_ = 2.50–9.45, *r* = 0.21–0.27, PD = 97.7–99.4%, ROPE = 14.6–5.4%). No significant mediation effects existed (Table 3).

Two of ten functional connections linked to aerobic fitness in the TEMP–TEMP cluster were correlated with PANSS-total, -positive and -negative scores as well as CGI score (BF_10range_ = 2.61–4.17, *r* = − 0.21 to − 0.24, PD = 98.2–98.6%, ROPE = 12.7–9.8%). All mediation effects were significant supporting the attenuating effect of aerobic fitness on total symptom severity mediated by functional connections between seeds from the temporal lobe (Table 3).

## Discussion

The present study was the first to explore the association between aerobic fitness and the whole-brain functional connectome in patients with schizophrenia while considering clinical and cognitive relevance of the identified fitness-connectivity relations.

First, we showed that higher patients’ aerobic fitness levels were associated with lower FC between the basal ganglia/thalamus and the midbrain/cerebellum network leading to lower psychopathological symptom severity. Second, FC between parts of the visual network and the right-lateralized FPN mediated the beneficial impact of aerobic fitness on verbal declarative memory. Third, higher patients’ aerobic fitness was accompanied by higher FC within the DMN, but clinical and cognitive relevance was lacking. Fourth, functional connections between cerebellar seeds and seeds from the subcortical nuclei as well as between seeds from the temporal lobe influenced the beneficial effect of aerobic fitness on total symptom severity. Fifth, FC between cerebellar seed connections mediated the positive influence of aerobic fitness on global cognition. Finally, functional connections between seeds from central cortical areas and from the limbic lobe drove the positive impact of aerobic fitness on emotion recognition.

Higher patients’ aerobic fitness levels are linked to lower FC between parts of the subcortical nuclei (basal ganglia, thalamus and amygdala [50]) and the cerebellum leading to attenuated total symptom severity. This is in line with previous studies suggesting a beneficial relation between aerobic fitness and different domains of psychiatric symptoms [22, 71, 72]. Within this association, our results are the first underlining the mediating role of FC between the subcortical nuclei and the cerebellum. Recent evidence proposes a state-independent cerebello-thalamo-cortical hyperconnectivity compared to healthy controls as a heritable neural signature in schizophrenia related to domains of positive symptomatology, especially disorganized thoughts and behavior [73, 74]. Based on the NMDA receptor hypofunction hypothesis of schizophrenia [75, 76], cerebello-thalamo-cortical hyperconnectivity is supposed to result from NMDA receptor deficits impeding the functioning of cortical parvalbumin-containing gamma-aminobutyric acid (GABA) interneurons which fail to inhibit pyramidal glutamatergic neurons eliciting upregulated FC within this circuitry [73, 77]. In line with the cognitive dysmetria theory of schizophrenia [78], the cerebello-thalamo-cortical hyperconnectivity may represent psychosis-related increased efforts in processing motion and cognition errors accurately [73]. Consequently, our findings could indicate that parts of the schizophrenia-specific cerebello-thalamo-cortical hyperconnectivity pattern are attenuated if patients have higher aerobic fitness levels leading to ameliorations in total symptom severity.

The beneficial impact of aerobic fitness on total symptom severity is also mediated by FC between temporal seeds as indicated by our results. An association between aerobic fitness and functional connections within temporal regions has already been proposed in healthy subjects [21]. Our finding supports this outcome and provides novel evidence on the mediating role of FC between temporal seeds. Specifically, the functional connections between the right Heschl’s gyrus and the left middle temporal gyrus and between the right superior temporal and the right middle temporal gyrus mediated the attenuating effect of aerobic fitness on total symptom severity. These regions are part of the auditory system and fulfil a broad range of different auditory and verbal tasks such as sound perception and recognition as well as language comprehension and production [79, 80]. In schizophrenia, functional deteriorations of the activation of the auditory system are consistently related to the severity of auditory hallucinations [81–84] and to disorganized speech [85] both reflecting positive symptoms. Functional overactivation of the middle and superior temporal gyri has been found in patients suffering from auditory hallucinations [83]. Accordingly, functional disconnections of the superior temporal gyrus have been related to the predisposition to develop auditory hallucinations [86]. Hence, it seems conceivable that the beneficial effect of aerobic fitness on total symptom severity is mediated by FC between auditory seed regions.

Furthermore, our findings suggest that patients’ aerobic fitness strengthened FC between cerebellar seeds leading to ameliorations in global cognition. Correspondingly, aerobic fitness and global cognition have been found to be correlated positively in patients with schizophrenia [87]. Simultaneously, recent findings in healthy subjects indicate that higher aerobic fitness levels are related to increased FC within the cerebellum [21]. In schizophrenia, hypoconnectivity patterns within the cerebellum have been reported [88]. Generally, the cerebellum—as part of the cortico-cerebellar-thalamic-cortical circuit—acts as a modulating system detecting patterns, changes and errors in motion and cognitive processes and providing adaptive neural feedback to cortical areas [89]. Multimodal cerebellar disturbances in schizophrenia are linked to deteriorations in multiple higher-order cognitive domains such as memory or attentional processes [78, 89]. Taken together, fitness-induced improvements in global cognition mediated by strengthened functional connections within the cerebellum seem plausible.

Finally, we observe a positive influence of aerobic fitness on emotion recognition capability mediated by FC between central and limbic seeds. A positive link between aerobic fitness and emotion recognition has recently been demonstrated in healthy participants [90]. We could replicate this finding in people with schizophrenia and provide new evidence on the mediating role of FC between the right Rolandic operculum and the bilateral posterior cingulate gyrus. The former integrates different kinds of sensory signals guiding interoceptive awareness and physical self-consciousness and is involved in emotion processing [91]. The posterior cingulate cortex represents the central node of the DMN facilitating internally directed cognition such as the retrieval of autobiographical memories, but is also supposed to regulate the focus of attention [92]. Consequently, it seems reasonable that the functional connections between the right Rolandic operculum and the bilateral posterior cingulate gyrus mediates the beneficial impact of aerobic fitness on emotion recognition.

Our exploratory examination comes along with a few limitations yielding important implications for future research: As indicated by the BFDA, the probability to detect a BF_10_ > 3 assuming a small population effect is only 30.6% using our study design (for details see suppl. S9). Furthermore, we do not correct for multiple comparisons. Because in Bayesian statistics no classical statistical test is performed resulting in a binary decision (effect vs. no effect), it is not common to correct the BF in case of a multiple test situation. Therefore, it is essential to consider the magnitude of the BF and the corresponding evidence level in favor of the alternative hypothesis instead of defining every BF > 3 as a robust effect. Importantly, in case of the mediation analysis, we do not correct the p values neither because we aim to detect even small effects. Future studies can build upon our preliminary findings using a hypothesis-driven approach based on an a-priori BFDA to ensure sufficient statistical power.

Further, the causal interpretations based on the results from mediation analysis in our cross-sectional study design have to be interpreted carefully. Although we control for age, sex, BMI, education years, disorder duration and chlorpromazine equivalents, we cannot rule out that other influencing variables exist, affecting our mediation analysis and leading to spurious interactions. Therefore, we interpret our preliminary findings in consideration of current literature knowledge on behavioral tasks of the ICNs and anatomical regions as well as on reported connectivity-fitness associations in other populations. Randomized-controlled intervention studies including an aerobic exercise program are needed to provide stable causal inferences concerning the FC-mediated effects of aerobic exercise on clinical and cognitive outcome.

Finally, we have no healthy control group available, but we still can draw first cautious conclusions on fitness-induced changes in FC leading to specific clinical or cognitive outcome. However, future studies should include a healthy control group and compare an aerobic exercise program to other types of physical activity interventions to verify possible compensatory effects and FC-mediated beneficial impacts of aerobic fitness on clinical and cognitive outcomes.

## Conclusion

To the best of our knowledge, our findings provide first insights into the role of fitness-induced adaptations of macro-scale FC patterns underlying benefits in symptomatology and cognition in people with schizophrenia. We emphasize that the results of this global exploratory analysis need further replication within a hypothesis-driven, randomized-controlled, interventional aerobic exercise study design.

## Supplementary Information

Below is the link to the electronic supplementary material.

Supplementary file1 (JPG 215 KB)

Supplementary file2 (JPEG 147 KB)

Supplementary file3 (JPEG 94 KB)

Supplementary file4 (JPEG 190 KB)

Supplementary file5 (JPEG 327 KB)

Supplementary file6 (JPEG 83 KB)

Supplementary file7 (JPEG 266 KB)

Supplementary file8 (JPEG 412 KB)

Supplementary file9 (JPEG 1249 KB)

Supplementary file10 (JPEG 1029 KB)

Supplementary file11 (JPEG 452 KB)

Supplementary file12 (JPEG 300 KB)

Supplementary file13 (JPEG 460 KB)

Supplementary file14 (JPEG 307 KB)

Supplementary file15 (DOCX 71 KB)

## Data Availability

Imaging data, results from the quality control and the Jupyter and R-scripts for the whole analysis as well as demographic, physical, clinical and cognitive data files are published on OSF (https://osf.io/tr3nx/?view_only=d2b15fb0503043328a7a27d0ba3a801f). Additional data can be made available upon request.

## Abbreviations

AAL: Automated anatomical labelling
ACME: Average causal mediation effect
B-CATS: Brief Cognitive Assessment Tool for Schizophrenia
BF_10_: Relative evidence for alternative hypothesis compared to null hypothesis
BFDA: Bayes factor design analysis
BIDS: Brain imaging data structure
BMI: Body-mass-index
BOLD: Blood oxygenation level dependent signal
CDSS: Calgary Depression Scale for Schizophrenia
CENTRAL–CENTRAL: Functional connections between seeds from central regions
CENTRAL–LIM: Functional connections between seeds from central regions and the limbic lobe
CEREB–CEREB: Functional connections between cerebellar seeds
CEREB–NUC: Functional connections between cerebellar seeds and seeds from subcortical nuclei
CGI: Clinical global impression
CI: Confidence interval
DICOM: Digital imaging and communications in medicine
DMN: Default mode network
DSM: Diagnostic and statistical manual of mental disorders
DSST: Digit symbol substitution test
DST: Digit span test
DV: Dependent variable
ICN: Intrinsic connectivity network
ESPRIT: Enhancing schizophrenia prevention and recovery through innovative treatments
Eff.: Effect (within mediation analysis)
EPI: Echo-planar imaging
fMRI: Functional magnetic resonance imaging
FPN: Fronto-parietal network
FWHM: Full width at half maximum
GABA: Gamma-aminobutyric acid
H_0_: Null hypothesis assuming that the true correlation equals zero
H_1_: Null hypothesis assuming that the true correlation does not equal zero
HDI: Highest density interval
ICA-AROMA: Automatic removal of motion artifacts based on independent component analysis
IV: Independent variable
LIM-TEMP: Functional connections between seeds from limbic and temporal lobe
MP-RAGE: T1-weighted magnetization prepared rapid gradient echo
NIFTI: Neuroimaging Informatics Technology Initiative
NMDA: *N*-Methyl-*D*-aspartate
OCC-TEMP: Functional connections between seeds from occipital and temporal lobe
*p*: Uncorrected *p* value
PANSS: Positive and Negative Syndrome Scale for Schizophrenia
PD: Probability of direction
ROPE: Region of practical equivalence
*r*: Pearson’s correlation coefficient
FC: Static functional connectivity
SN: Salience network
TEMP-TEMP: Functional connections between seeds from temporal lobe
TMT-A: Trail making test A
TMT-B: Trail making test B
VLMT: Verbal learning and memory test
