# Supplementary figures and images for: Association between aerobic fitness and the functional connectome in patients with schizophrenia
Source: Eur Arch Psychiatry Clin Neurosci. 2022 Apr 30;272(7):1253–72. doi: 10.1007/s00406-022-01411-x (PMC9508005; doi:10.1007/s00406-022-01411-x)

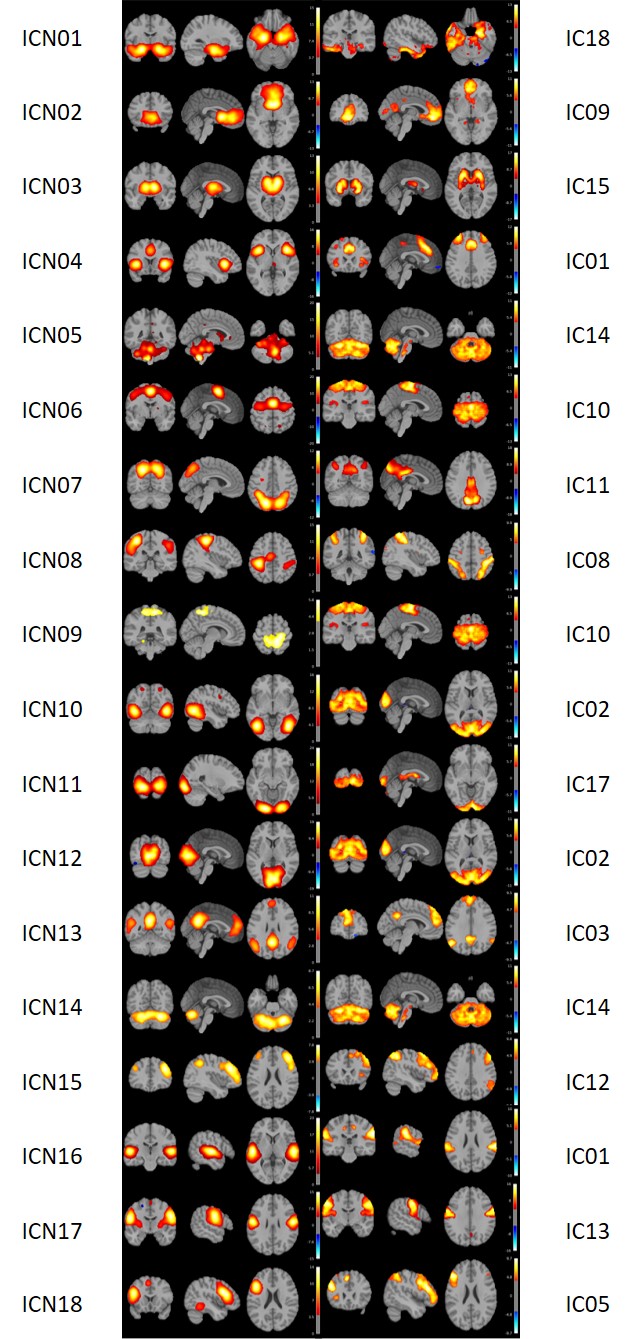

Supplement: Supplementary file 1 — Supplementary file1 (JPG 215 KB) [file 406_2022_1411_MOESM1_ESM.jpg]

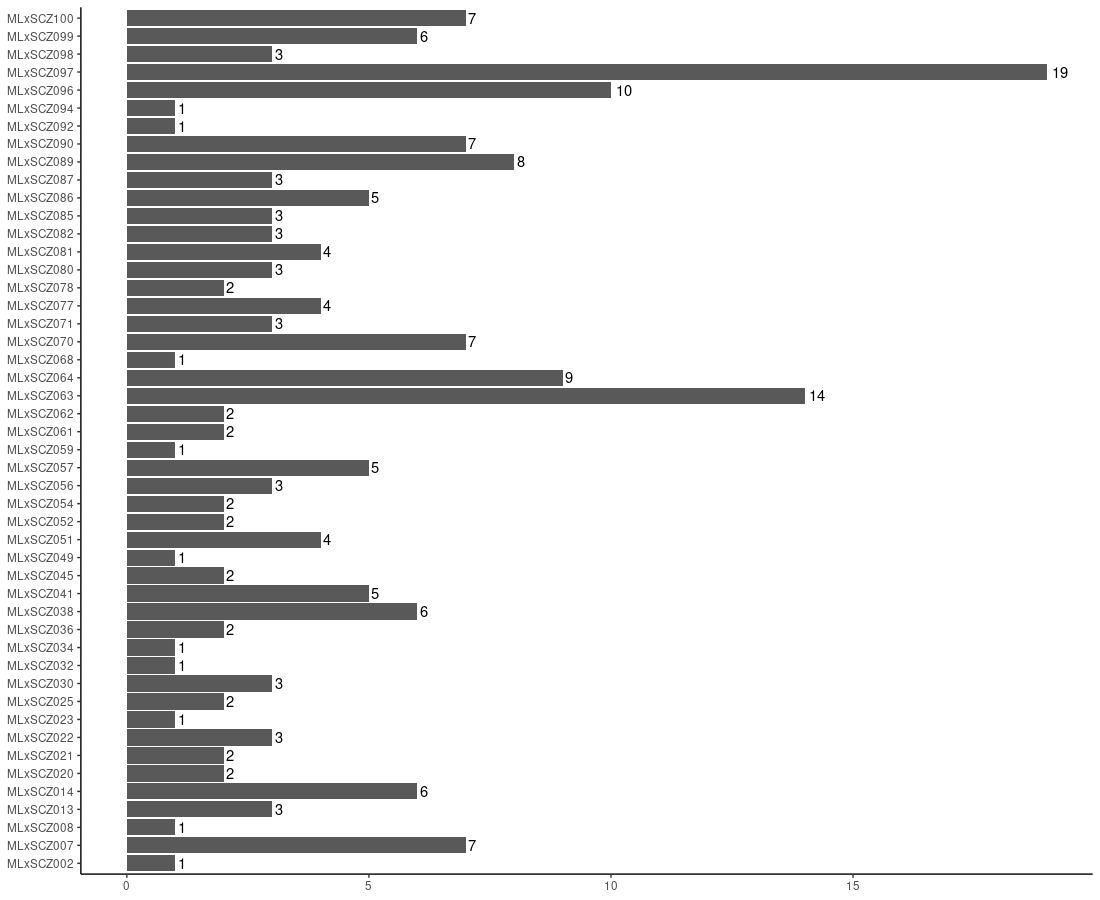

Supplement: Supplementary file 2 — Supplementary file2 (JPEG 147 KB) [file 406_2022_1411_MOESM2_ESM.jpeg]

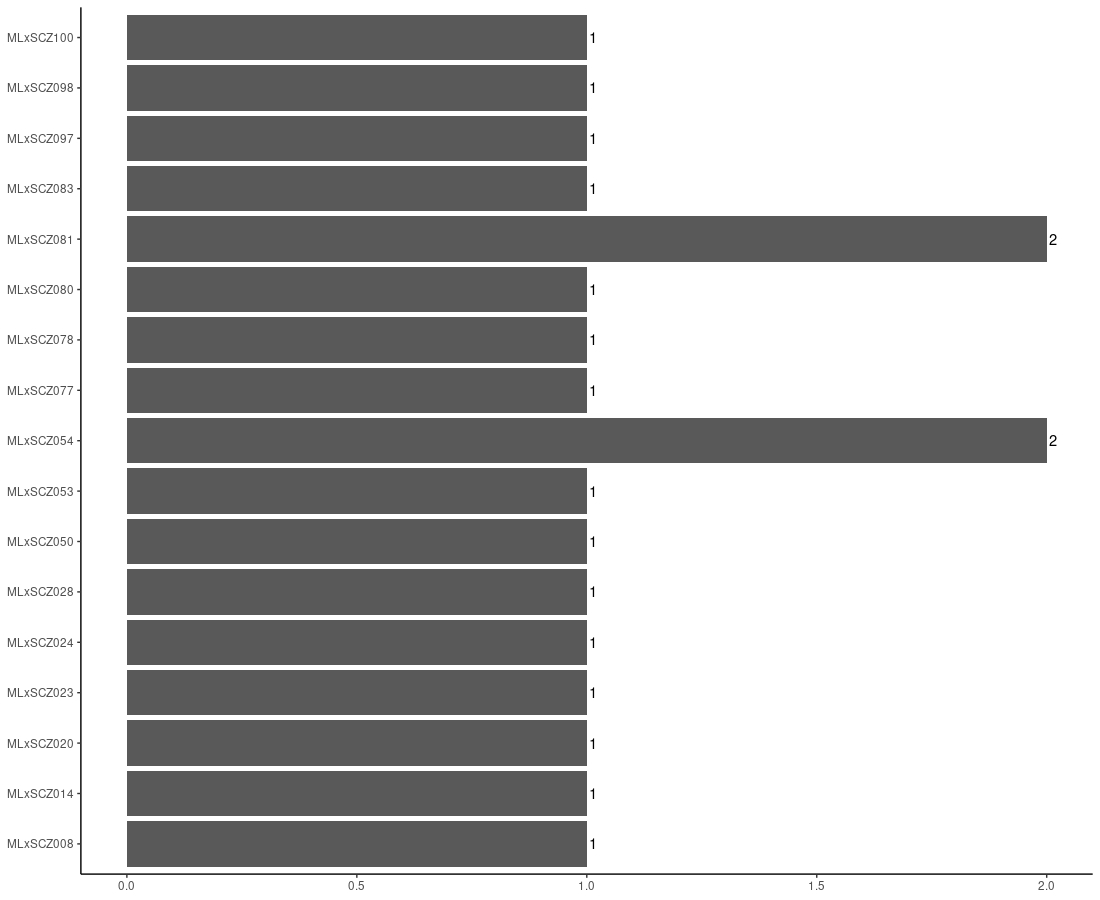

Supplement: Supplementary file 3 — Supplementary file3 (JPEG 94 KB) [file 406_2022_1411_MOESM3_ESM.jpeg]

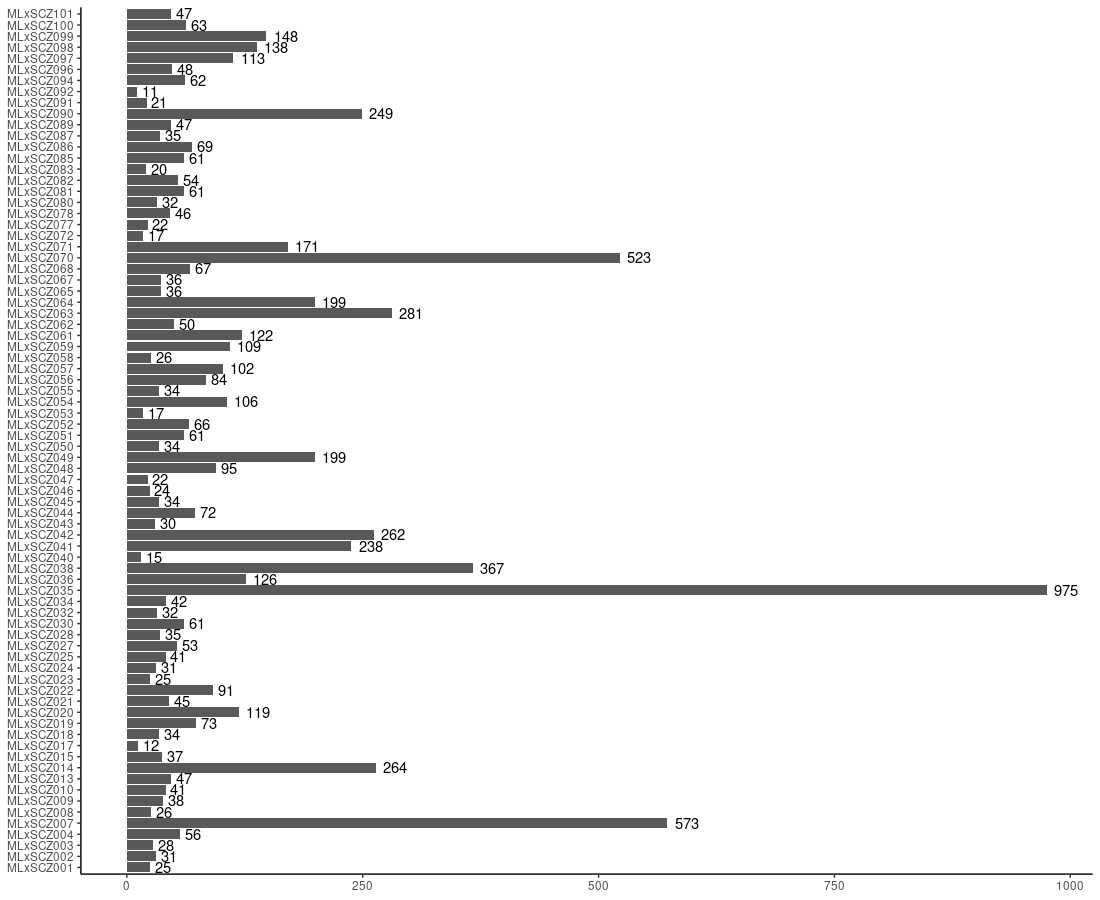

Supplement: Supplementary file 4 — Supplementary file4 (JPEG 190 KB) [file 406_2022_1411_MOESM4_ESM.jpeg]

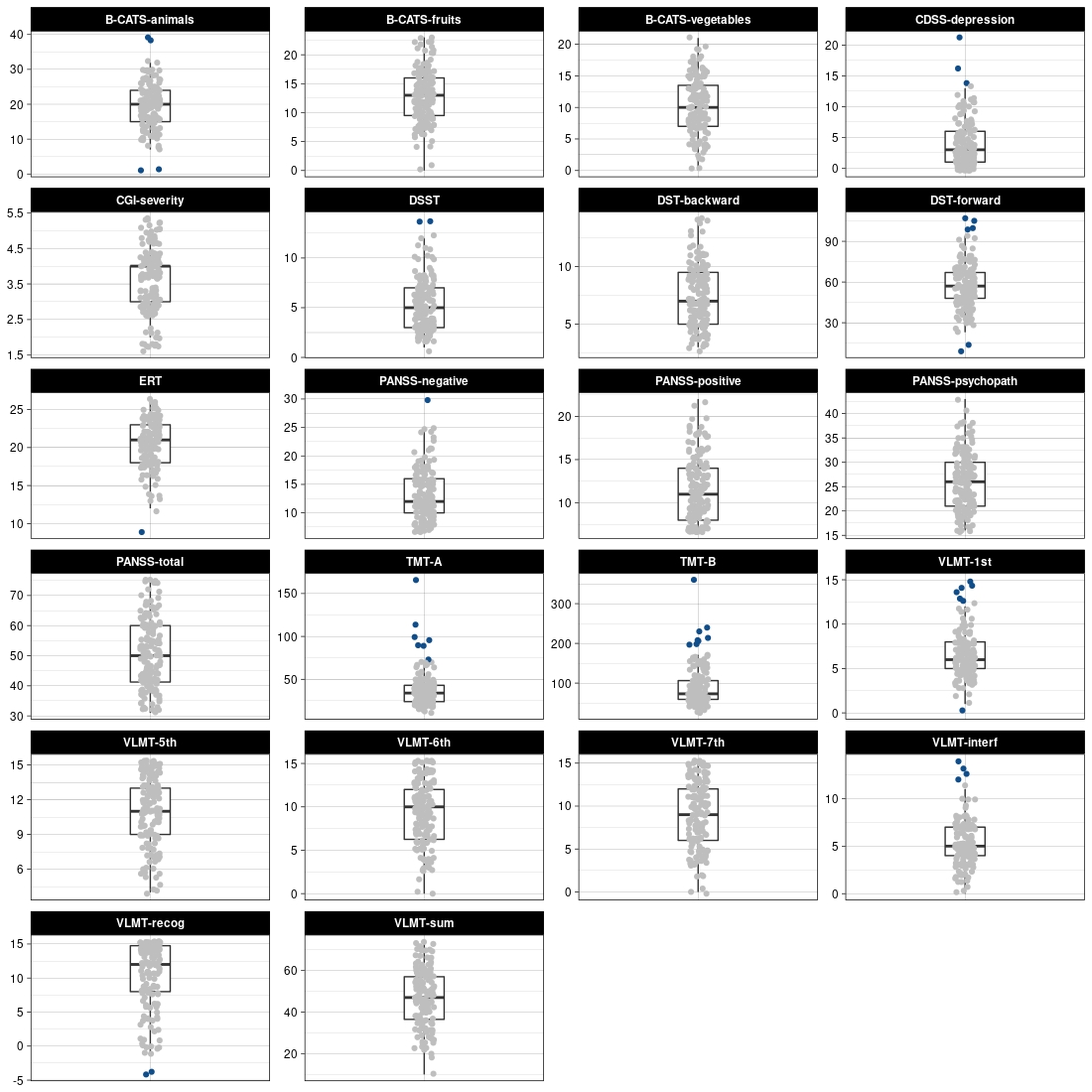

Supplement: Supplementary file 5 — Supplementary file5 (JPEG 327 KB) [file 406_2022_1411_MOESM5_ESM.jpeg]

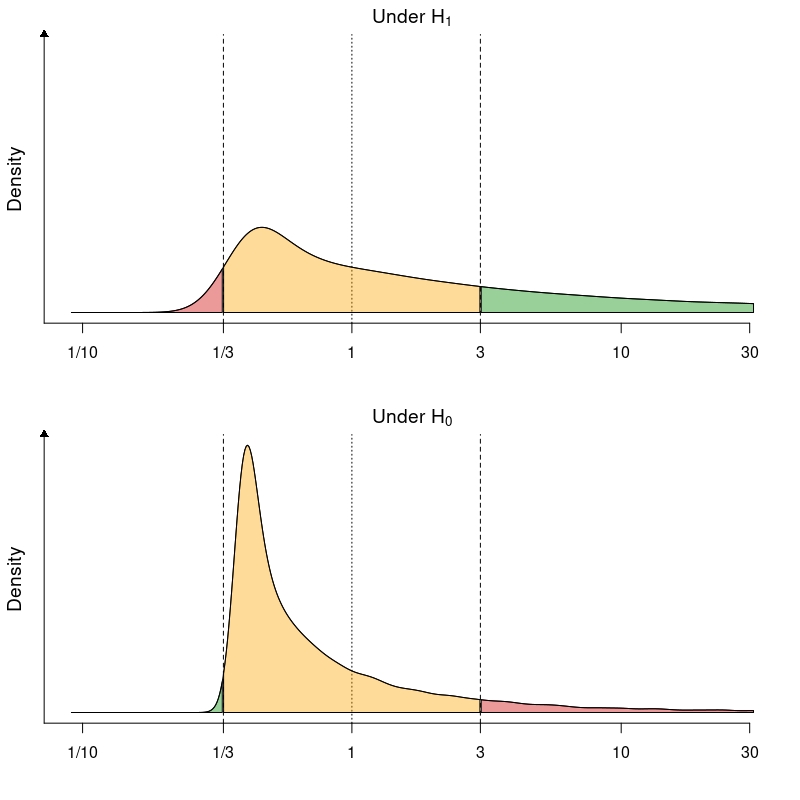

Supplement: Supplementary file 6 — Supplementary file6 (JPEG 83 KB) [file 406_2022_1411_MOESM6_ESM.jpeg]

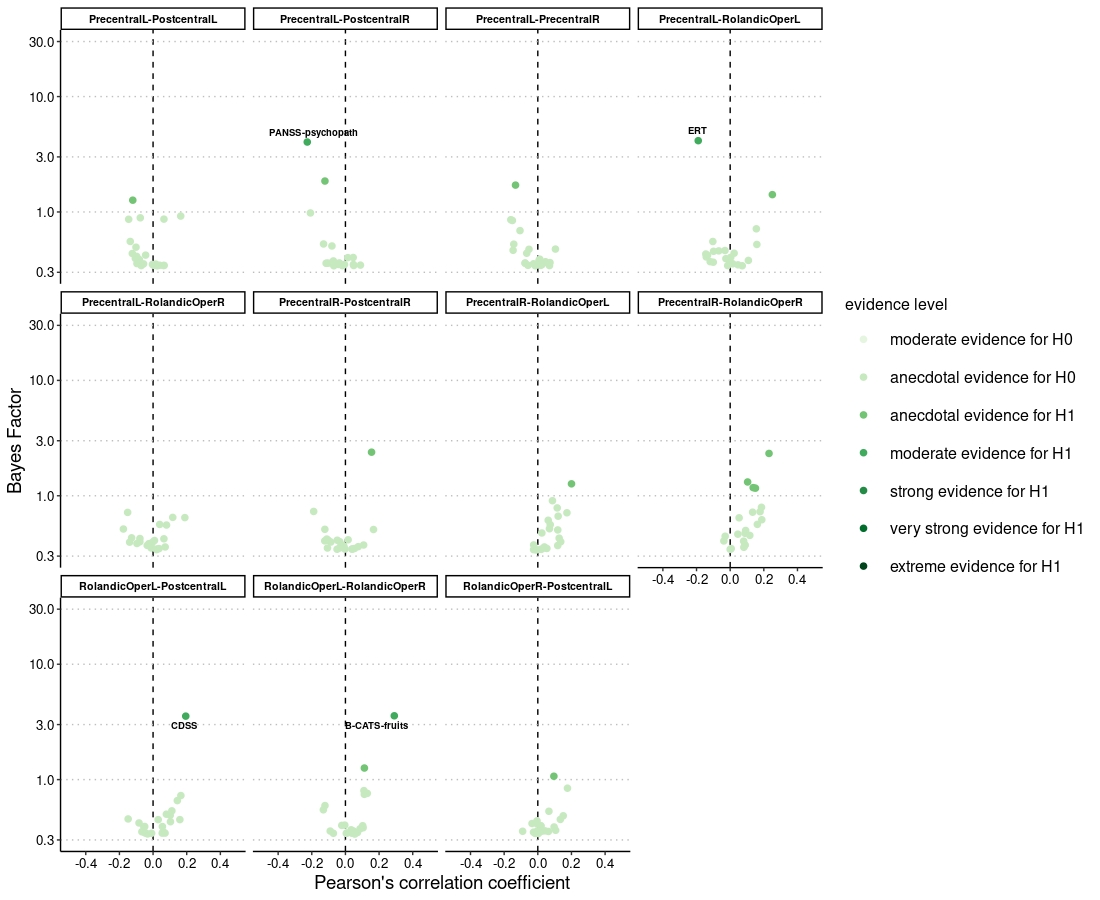

Supplement: Supplementary file 7 — Supplementary file7 (JPEG 266 KB) [file 406_2022_1411_MOESM7_ESM.jpeg]

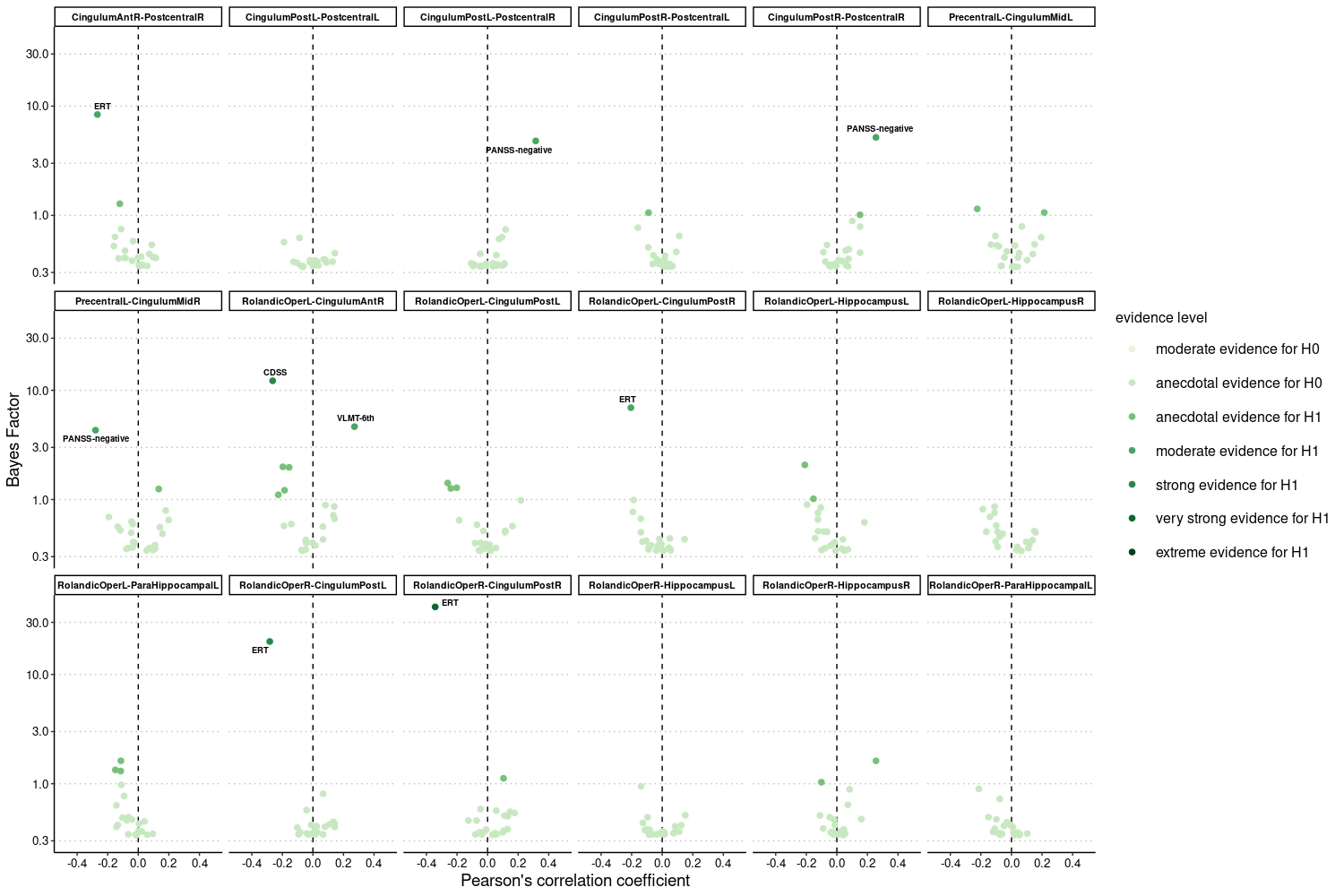

Supplement: Supplementary file 8 — Supplementary file8 (JPEG 412 KB) [file 406_2022_1411_MOESM8_ESM.jpeg]

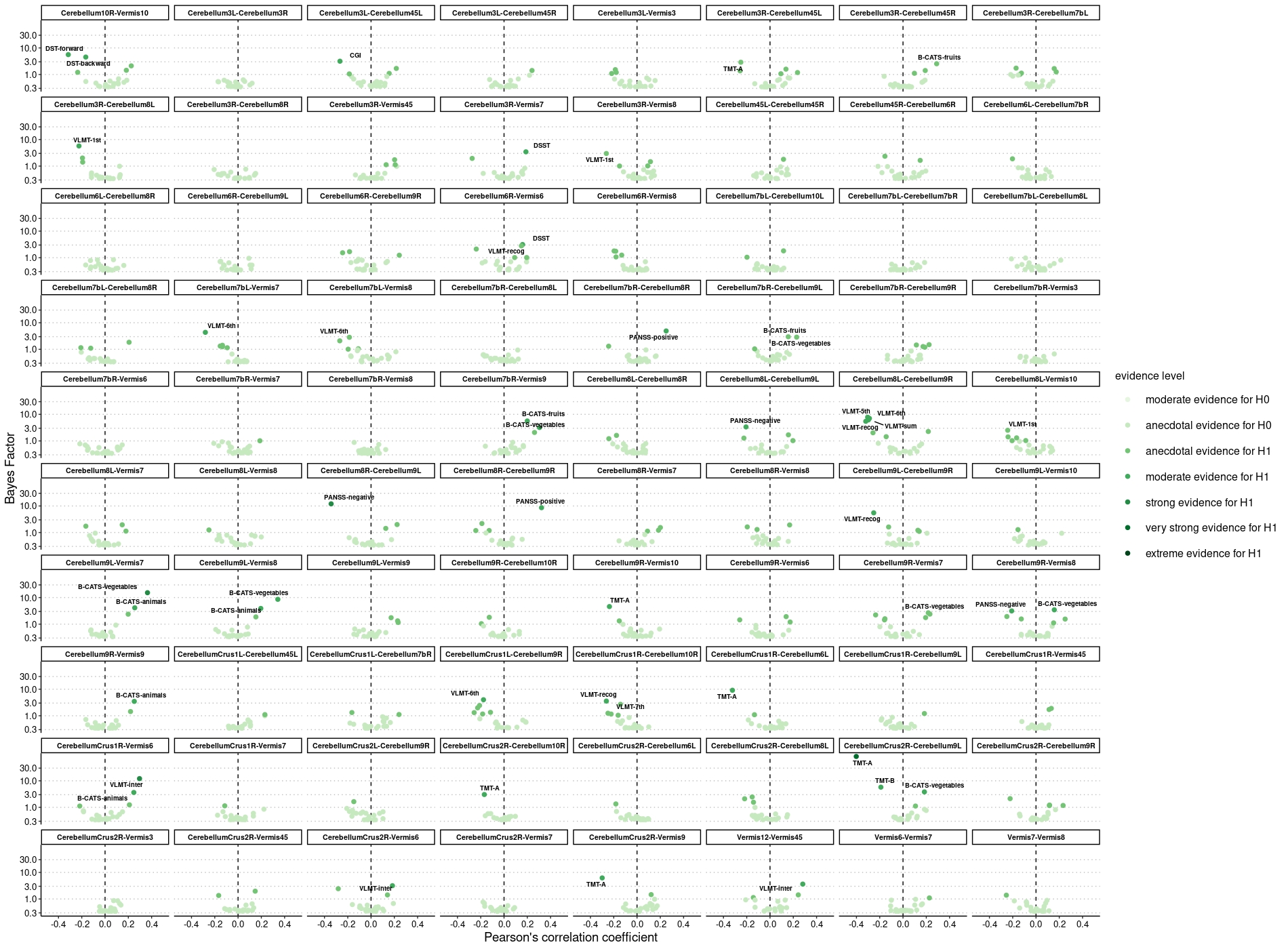

Supplement: Supplementary file 9 — Supplementary file9 (JPEG 1249 KB) [file 406_2022_1411_MOESM9_ESM.jpeg]

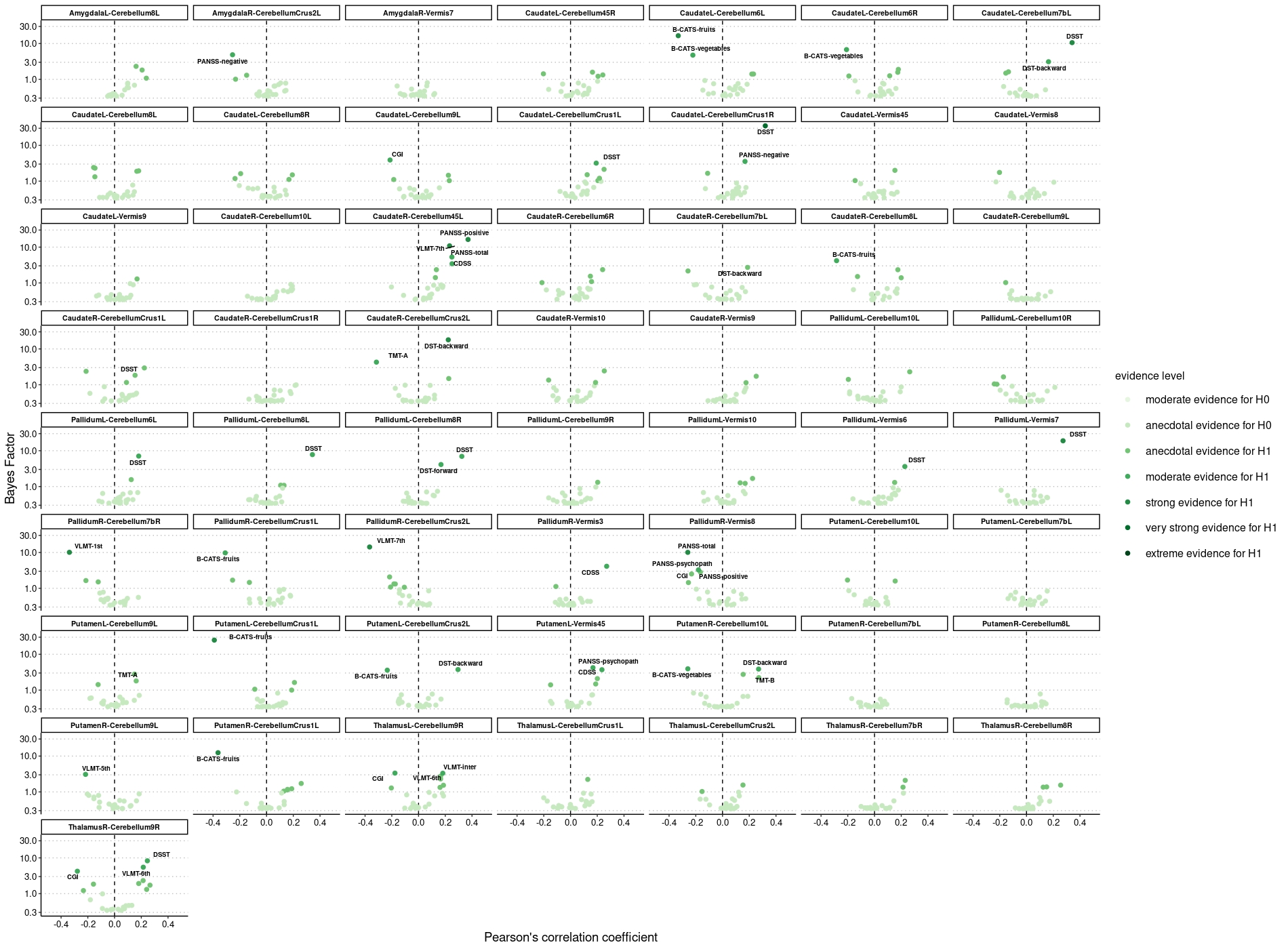

Supplement: Supplementary file 10 — Supplementary file10 (JPEG 1029 KB) [file 406_2022_1411_MOESM10_ESM.jpeg]

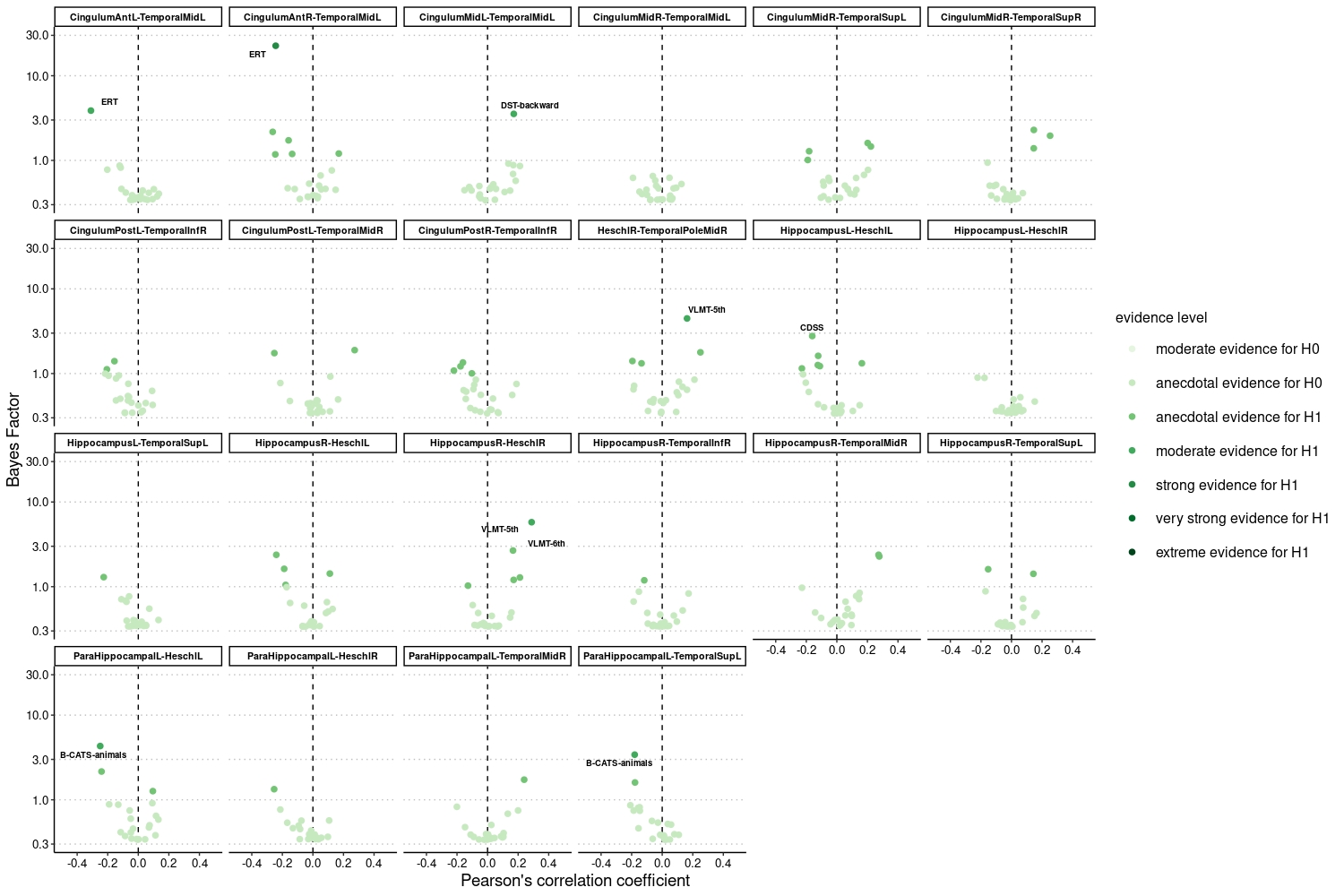

Supplement: Supplementary file 11 — Supplementary file11 (JPEG 452 KB) [file 406_2022_1411_MOESM11_ESM.jpeg]

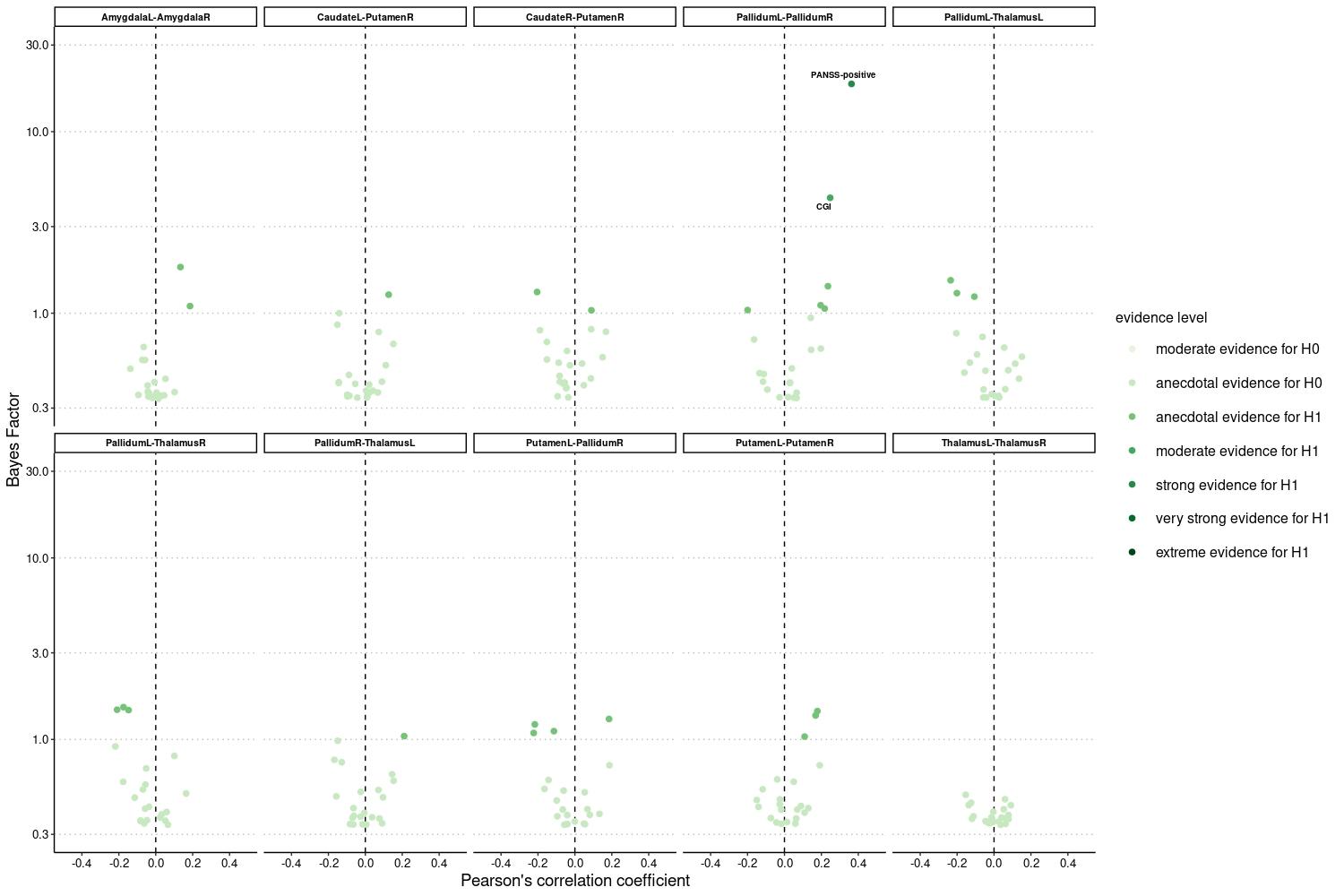

Supplement: Supplementary file 12 — Supplementary file12 (JPEG 300 KB) [file 406_2022_1411_MOESM12_ESM.jpeg]

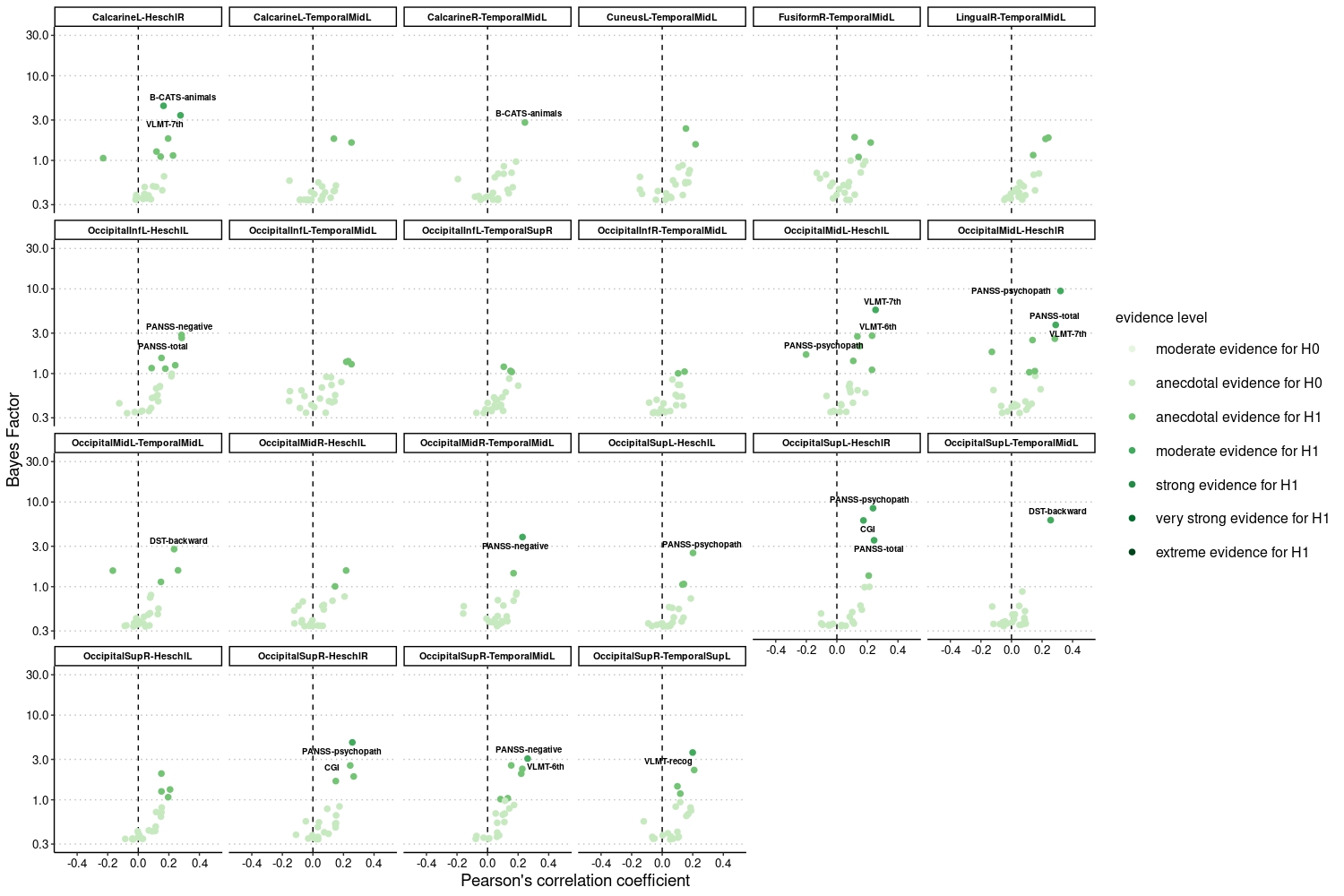

Supplement: Supplementary file 13 — Supplementary file13 (JPEG 460 KB) [file 406_2022_1411_MOESM13_ESM.jpeg]

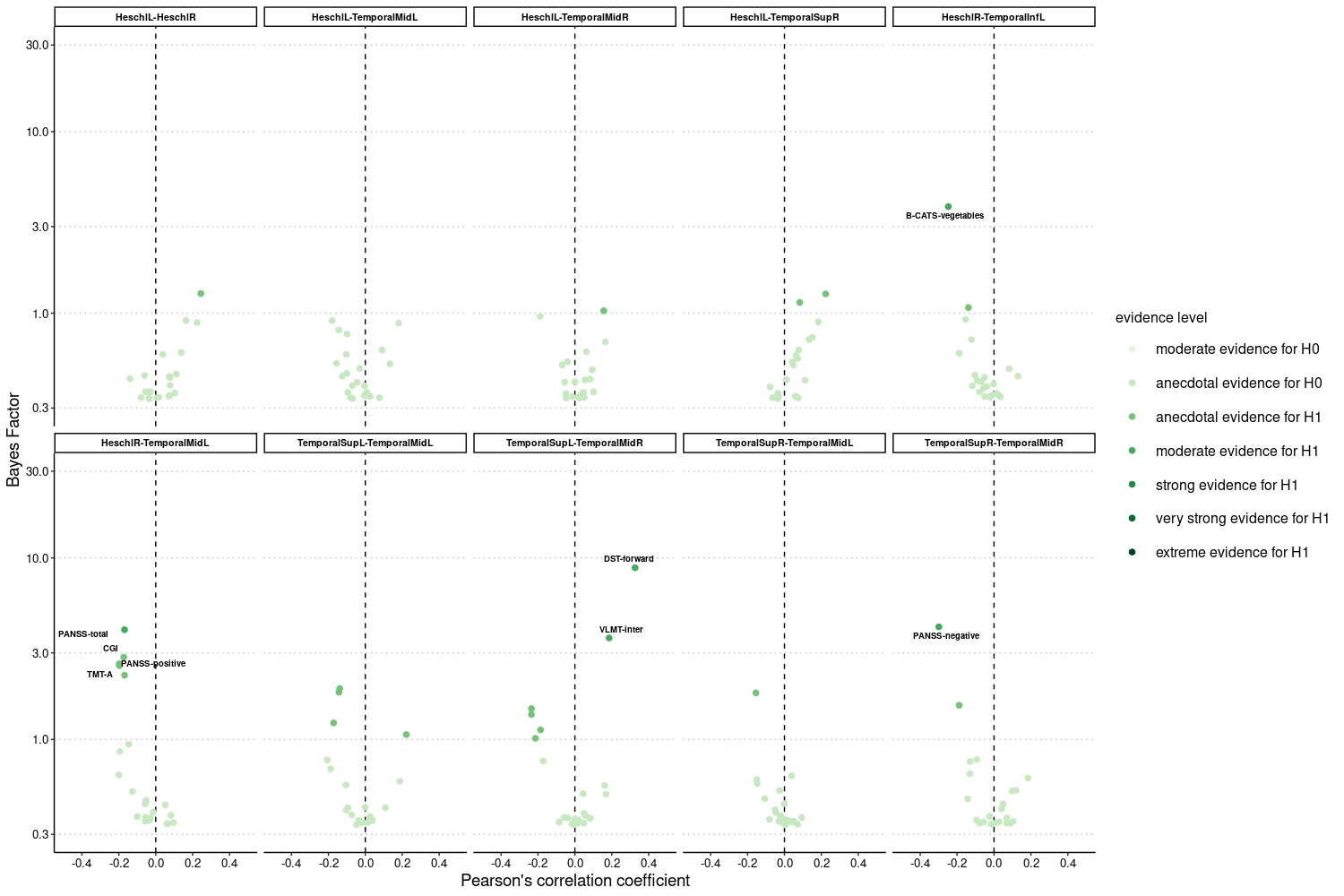

Supplement: Supplementary file 14 — Supplementary file14 (JPEG 307 KB) [file 406_2022_1411_MOESM14_ESM.jpeg]
