# Supplementary material for: Association between aerobic fitness and the functional connectome in patients with schizophrenia
Source: Eur Arch Psychiatry Clin Neurosci. 2022 Apr 30;272(7):1253–72. doi: 10.1007/s00406-022-01411-x (PMC9508005; doi:10.1007/s00406-022-01411-x)
Supplement: Supplementary file 15 — Supplementary file15 (DOCX 71 KB) [file 406_2022_1411_MOESM15_ESM.docx]

**Supplemental materials**

Association between aerobic fitness and the functional connectome in patients with schizophrenia

Lukas Röll^1,9^*, Isabel Maurus^1^, Daniel Keeser^1,2,9^, Temmuz Karali^1,2,9^, Boris Papazov^2^, Alkomiet Hasan^3^, Andrea Schmitt^1,4^, Irina Papazova^3^, Moritz Lembeck^1^, Dusan Hirjak^5^, Eliska Sykorova^5^, Cristina E. Thieme^5^, Susanne Münz^1^, Valentina Seitz^1^, David Greska^1^, Mattia Campana^1^, Elias Wagner^1^, Lisa Löhrs^1^, Sophia Stöcklein^2^, Birgit Ertl-Wagner^2,10^, Johannes Pömsl^6^, Astrid Roeh^3^, Berend Malchow^7^, Katriona Keller-Varady^8^, Andreas Meyer-Lindenberg^5^, and Peter Falkai^1^

^1^Department of Psychiatry and Psychotherapy, University Hospital, LMU Munich, Munich, Germany

^2^Department of Radiology, University Hospital, LMU Munich, Munich, Germany

^3^Department of Psychiatry and Psychosomatics of the University Augsburg, Bezirkskrankenhaus Augsburg, University of Augsburg, Augsburg, Germany

^4^Laboratory of Neuroscience (LIM27), Institute of Psychiatry, University of Sao Paulo, São Paulo, Brazil

^5^Central Institute of Mental Health, Medical Faculty Mannheim, Heidelberg University, Germany

^6^Department of Psychiatry and Psychotherapy, Medical Faculty, Technical University of Munich, University hospital ´Klinikum rechts der Isar´, Munich, Germany

^7^Department of Psychiatry and Psychotherapy, University Hospital Göttingen, Göttingen, Germany

^8^Hannover Medical School, Institute of Sports Medicine, Hannover, Germany

^9^NeuroImaging Core Unit Munich (NICUM), University Hospital LMU, Munich, Germany

^10^Division of Neuroradiology, Department of Diagnostic Imaging, The Hospital for Sick Children, Toronto, Canada

*Correspondence concerning this article should be addressed to Lukas Röll, Department of Psychiatry and Psychotherapy, University Hospital, LMU Munich, Nussbaumstrasse 7, 80336 Munich, Germany. Email: [Lukas.Roell@med.uni-muenchen.de](mailto:Lukas.Roell@med.uni-muenchen.de)

**S1: scanning parameters**

Table S1 illustrates the scanning parameters at study sites Munich and Mannheim. At study site Munich a 20-channel head coil was utilized, whereas in Mannheim a 32-channel multi-array head coil was used.

**Tab. S1**

Scanning parameters

| **site** | **sequence** | **FoV** | **resolution** | **TR** | **TE** | **TI** | **FA** | **slices** | **timepoints** |
| --- | --- | --- | --- | --- | --- | --- | --- | --- | --- |
|  |  |  |  |  |  |  |  |  |  |
| Munich | MP-RAGE | 240 mm | 0.8 × 0.8 × 0.8 mm³ | 2060 ms | 2.17 ms | 1040 ms | 12° | 256 | - |
| Munich | EPI | 216 mm | 3.0 × 3.0 × 3.0 mm³ | 3000 ms | 30 ms | - | 85° | 45 | 124 |
|  |  |  |  |  |  |  |  |  |  |
| Mannheim | MP-RAGE | 256 mm | 1.0 × 1.0 × 1.0 mm³ | 2530 ms | 3.8 ms | 1100 ms | 7° | 176 | - |
| Mannheim | EPI | 192 mm | 3.0 × 3.0 × 3.0 mm³ | 1790 ms | 28 ms | - | 76° | 34 | 230 |

*Note.* Sequence = type of scanning sequence, FoV = field of view, resolution = voxel size, TR = Time of repetition, TE = echo time, TI = inversion time, FA = flip angle, slices = number of acquired slices, MP-RAGE = T1-weighted magnetization prepared rapid gradient echo, EPI = echo planar imaging.

**S2: Quality control of fMRI data and decisions on data exclusion**

The command and the results from the automated quality control software MRIQC will be published on OSF. We inspected all images visually and documented important quality metrics before and after pre-processing, after smoothing and after denoising. Particularly, we evaluated the signal-to-noise ratio (SNR) after pre-processing and smoothing, framewise displacement (FD) and DVARS after pre-processing as well as the correlations between FD- and BOLD-timeseries and correlations between mean FD and static functional connectivity (FC) values across all participants after denoising. Participants were excluded in the following scenarios: SNR < 30 after smoothing, FD > 0.5 after pre-processing, DVARS > 50 after pre-processing. Using the correlations between FD- and BOLD-timeseries and mean FD and FC values, we evaluated if head motions were still linked to the BOLD signal and FC after denoising. The corresponding frequent cases were excluded as well. The detailed documentation sheets of the quality control will be published on OSF. The following nine subjects were excluded due to lacking image quality: MLxSCZ006, MLxSCZ016, MLxSCZ029, MLxSCZ060, MLxSCZ066, MLxSCZ069, MLxSCZ079, MLxSCZ088 and MLxSCZ095. In case of further nine subjects, resting-state fMRI data was lacking: MLxSCZ011, MLxSCZ012, MLxSCZ031, MLxSCZ033, MLxSCZ073, MLxSCZ074, MLxSCZ075, MLxSCZ076 and MLxSCZ093.

**S3: Pre-processing of fMRI data**

The command from the pre-processing pipeline FMRIPREP will be published under https://osf.io/tr3nx/?view_only=d2b15fb0503043328a7a27d0ba3a801f. Within FMRIPREP the following steps were executed: T1-weighted (T1w) volumes were corrected for INU (intensity non-uniformity) using *N4BiasFieldCorrection v2.1.0* [1] and skull-stripped using *antsBrainExtraction.sh v2.1.0* (OASIS template) [2]. Brain-extracted images were spatially normalized to the brain-extracted ICBM 152 Nonlinear Asymmetrical template version 2009c [3] using nonlinear registration within the *antsRegistration* tool of ANTs v2.1.0 [4]. Brain tissue segmentation was performed using *fast* from FSL v5.0.9 [5, 6]. Resting-state fMRI data was slice time corrected with *3dTshift* from AFNI v16.2.07 [7] and motion corrected utilizing *mcflirt* from FSL v5.0.9 [6, 8]. Distortion correction was performed by co-registering the fMRI image to the same-subject T1w image with intensity inversion [9, 10] constrained by an average fieldmap template [11] implemented in *antsRegistration* [4]. Thereafter, co-registration to the corresponding T1w image using boundary-based registration [12] with twelve degrees of freedom was executed with *flirt* from FSL v5.0.9 [6, 8, 13]. Motion correcting transformations, field distortion correcting warp, BOLD-to-T1w transformation and T1w-to-template (MNI152NLin2009cAsym at 2mm resolution) warp were administered in one step using *antsApplyTransforms* from ANTs v2.1.0 [2]. FD [14] was computed for every functional run using the implementation in Nipype [15]. Automatic Removal Of Motion Artifacts based on independent component analysis (ICA-AROMA) was utilized to extract aggressive noise regressors [16]. For more details of the fmriprep pipeline see <https://fmriprep.readthedocs.io/en/stable/workflows.html>.

**S4: Post processing and ICN-IC assignment based on fslcc and visual inspection**

The post-processing workflow including the between-ICN, within-ICN and the seed-based connectivity approach is visualized in figure S4.1. The two ICN-approaches end with the ICN-IC assignments. For this, every site-specific independent component (IC) was matched to the corresponding intrinsic connectivity network (ICN) proposed by Laird et al. [17] using fslcc. If an IC correlated the highest with an ICN, the IC was supposed to represent this ICN. Sometimes the same IC was assigned to multiple ICNs (e.g. IC01 to ICN04 and ICN16 or IC14 to ICN05 and ICN14 in the sample from Munich). In those ambiguous cases we searched for an alternative IC that correlated similarly with the corresponding ICN and visualized it in order to figure out if the alternative IC fits better to the original ICN despite its slightly lower correlation coefficient. If a reasonable alternative IC was available, we corrected the assignment (e.g. IC01 replaced by IC07 reflecting ICN04 in the sample from Munich), if not we assumed the IC to summarize multiple ICNs (e.g. IC14 reflecting ICN05 and ICN 14 in the Munich sample). Table S4 displays all original and alternative ICN-IC-assignments including the correlation coefficients. Figure S4.2 and S4.3 visualize all ICs from Munich and Mannheim and the original ICNs proposed by Laird et al. [17].

**Tab. S4**

ICN-IC assignments

| **site** | **ICN** | **IC** | **r** | **IC alternative** | **r alternative** | **note** |
| --- | --- | --- | --- | --- | --- | --- |
|  |  |  |  |  |  |  |
| Munich | ICN01 | IC18 | 0.4928 | - | - | - |
|  | ICN02 | IC09 | 0.49081 | - | - | - |
|  | ICN03 | IC15 | 0.58286 | - | - | - |
|  | ICN04 | IC01 | 0.36343 | IC07 | 0.35645 | assignment corrected |
|  | ICN05 | IC14* | 0.52369 | - | - | - |
|  | ICN06 | IC10* | 0.32919 | - | - | - |
|  | ICN07 | IC11 | 0.41703 | - | - | - |
|  | ICN08 | IC08 | 0.33624 | - | - | - |
|  | ICN09 | IC10* | 0.59464 | - | - | - |
|  | ICN10 | IC02* | 0.36856 | - | - | - |
|  | ICN11 | IC17 | 0.50908 | - | - | - |
|  | ICN12 | IC02* | 0.59015 | - | - | - |
|  | ICN13 | IC03 | 0.4121 | - | - | - |
|  | ICN14 | IC14* | 0.70092 | - | - | - |
|  | ICN15 | IC12 | 0.44606 | - | - | - |
|  | ICN16 | IC01 | 0.30458 | - | - | - |
|  | ICN17 | IC13 | 0.62558 | - | - | - |
|  | ICN18 | IC05 | 0.47167 | - | - | - |
|  |  |  |  |  |  |  |
| Mannheim | ICN01 | IC18 | 0.34232 | - | - | - |
|  | ICN02 | IC12 | 0.50994 | - | - | - |
|  | ICN03 | IC20 | 0.29218 | - | - | - |
|  | ICN04 | IC08 | 0.30963 | - | - | - |
|  | ICN05 | IC18 | 0.26184 | IC15* | 0.25585 | assignment corrected |
|  | ICN06 | IC14* | 0.25101 | - | - | - |
|  | ICN07 | IC11 | 0.36817 | - | - | - |
|  | ICN08 | IC10 | 0.35533 | - | - | - |
|  | ICN09 | IC14* | 0.51198 | - | - | - |
|  | ICN10 | IC09* | 0.40174 | - | - | - |
|  | ICN11 | IC09* | 0.35109 | - | - | - |
|  | ICN12 | IC06 | 0.69352 | - | - | - |
|  | ICN13 | IC01 | 0.44819 | - | - | - |
|  | ICN14 | IC15* | 0.50213 | - | - | - |
|  | ICN15 | IC02 | 0.44692 | - | - | - |
|  | ICN16 | IC16* | 0.23827 | - | - | - |
|  | ICN17 | IC16* | 0.45273 | - | - | - |
|  | ICN18 | IC04 | 0.40118 | - | - | - |

*Note.* ICN = intrinsic connectivity network based on Laird et al. [17], IC = sample-specific independent component, r = correlation between ICN and IC, IC alternative = alternative IC with similar correlation and better visual match, r alternative = correlation between ICN and alternative IC, * = ICs that were assigned to multiple ICNs and could not be reassigned properly.

**Fig. S4.1**

Visualization of ICNs and ICs from study site Munich

*Note.* ICNs from Laird et al. are displayed on the left. Sample-specific ICs from study site Munich on the right. Both are mapped onto the MNI152NLin2009cAsym standard space with a resolution of 2 mm in neurological convention. Color mapping is not standardized across ICNs and ICs.

**Fig. S4.2**

Visualization of ICNs and ICs from study site Mannheim

*Note.* ICNs from Laird et al. are displayed on the left. Sample-specific ICs from study site Mannheim on the right. Both are mapped onto the MNI152NLin2009cAsym standard space with a resolution of 2 mm in neurological convention. Color mapping is not standardized across ICNs and ICs.

**S5: Details on cognitive test batteries**

During the TMT-A subjects had to connect numbers from 1 to 25 in ascending order as fast and accurate as possible without lifting the pencil from the sheet of paper. In TMT-B participants had to connect numbers and letters alternately in the following order until number 13 was reached: 1-A-2-B-3-C-4-D-5-E-6-F-7-G-8-H-9-I-10-J-11-K-12-L-13. The time needed in seconds was measured.

The task in the category naming part of the B-CATS was to name as many animals (B-CATS-animals), fruits (B-CATS-fruits) and vegetables (B-CATS-vegetables) as possible within one minute for each category. The number of correctly named animals, fruits or and vegetables reflected the achieved score.

In the DSST the subjects were asked to translate as many numbers as possible within 90 seconds into written symbols based on a predefined coding scheme. The number of correctly translated numbers was assessed.

During the DST-forward the experimenter read digit rows of increasing lengths that the subject had to repeat verbally in the same order. The test was stopped if the participant failed twice within the same “row length category”. DST-backward worked analogously except that the subjects had to repeat the digits in reverse order. The number of correct trials was counted in both versions.

Within the VLMT the experimenters read a list of 15 words and the participants had to remember as many words as possible in arbitrary order. This procedure was repeated five times in a row (VLMT-1^st^ to VLMT-5^th^) and the sum of correctly remembered words across the five trials was computed (VLMT-sum). After the fifth trial, an interference list of 15 different words was read and the subjects had to name as many words from this new list as possible (VLMT-inter). Thereafter, the subject was asked to remember as many words as possible from the first list (VLMT-6^th^) without repeating it again. After a 20 minutes delay in which other cognitive tests were executed, the participants had to remember as many words as possible from the first list again (VLMT-7^th^). Finally, the experimenters read 50 words including the ones from the first list and interference trial and the subjects had to decide if the corresponding word was part of the first list (VLMT-recog). The number of correctly remembered or recognized words in each trial was counted resulting in seven different VLMT-scores.

During the ERT emotional faces illustrating anger, fear, happiness, disgust, surprise, sadness or neutrality were presented to the subjects who had to recognize the correct emotion. The number of correctly recognized emotions was counted.

**S6: Decisions on second data exclusion of fMRI and clinical/cognitive data**

Aiming to detect prominent outliers, we counted the cases across all functional connections of the three approaches (between-ICN, within-ICN and seed-based) in which a subject was identified as an outlier. As indicated by figure S6.1, S6.2 and S6.3 subjects MLxSCZ007, MLxSCZ035, MLxSCZ038, MLxSCZ063, MLxSCZ070 and MLxSCZ070 reflected the most frequent outliers within the three analysis approaches, but the corresponding quality of the images was sufficient. Hence, those subjects were included in the final analysis.

Figure S6.4 visualizes the distributions of all clinical and cognitive test scores across the whole sample prior to outlier exclusion. Every raw data distribution was inspected visually in order to identify outliers. The latter were evaluated in more detail aiming to decide if the corresponding value was plausible or not. This evaluation process depended on the actual clinical or cognitive score. Regarding the cognitive test batteries, we excluded values reflecting very low performances and probably biased by lacking motivation. For instance, a few subjects were only able to name three or less animals, fruits or vegetables within one minute or needed more than 150 and 300 seconds to complete the TMT-A and TMT-B, respectively. Since the probability that these bad performances are caused by lacking motivation rather than by real cognitive impairments is very high, we decided to exclude these values by transforming them to missing values. In case of PANSS-negative and CDSS, we excluded two outliers because they deviated essentially from the remaining subjects.

We decided to set the following thresholds: B-CATS scores < 4, VLMT-recog < 0, DSST score < 15, TMT-A score < 150, TMT-B score < 300, PANSS-negative > 25, CDSS > 20. We emphasize that these thresholds are specific to the raw data distributions in our sample. The number of invalid and missing values differed across the clinical and cognitive test batteries. Tab. S6 illustrates the final sample sizes in each statistical analysis.

Importantly, we checked the influence of different outlier exclusion strategies on the results and did not observe large deviations which would have led to different conclusions.

**Fig. S6.1**

Counts of outliers per subject.

*Note.* The number of outlier values across all between-ICN connections per subject are displayed.

**Fig. S6.2**

Counts of outliers per subject*.*

*Note.* The number of outlier values across all within-ICN connections per subject are displayed.

**Fig. S6.3**

Counts of outliers per subject*.*

*Note.* The number of outlier values across all seed connections per subject are displayed.

**Fig. S6.4**

Distributions of clinical and cognitive raw scores*.*

*Note.* The raw data distributions of all clinical and cognitive test scores prior to outlier exclusion are shown. Blue dots reflect outliers.

**Tab. S6**

Sample sizes in the different statistical approaches

| Statistical analysis | Variable 1 | Mediator | Variable 2 | N |
| --- | --- | --- | --- | --- |
|  |  |  |  |  |
| Bayesian partial correlations | Aerobic fitness | - | Functional connections | 58 |
|  |  |  |  |  |
| Bayesian partial correlations | Functional connections | - | PANSS-positive | 79 |
|  | Functional connections | - | PANSS-negative | 78 |
|  | Functional connections | - | PANSS-psychopath | 79 |
|  | Functional connections | - | PANSS-total | 79 |
|  | Functional connections | - | CDSS | 79 |
|  | Functional connections | - | CGI | 79 |
|  | Functional connections | - | B-CATS-animals | 76 |
|  | Functional connections | - | B-CATS-fruits | 76 |
|  | Functional connections | - | B-CATS-vegetables | 72 |
|  | Functional connections | - | DSST | 77 |
|  | Functional connections | - | DST-forward | 78 |
|  | Functional connections | - | DST-backward | 79 |
|  | Functional connections | - | TMT-A | 78 |
|  | Functional connections | - | TMT-B | 77 |
|  | Functional connections | - | VLMT-1^st^ | 77 |
|  | Functional connections | - | VLMT-5^th^ | 77 |
|  | Functional connections | - | VLMT-sum | 77 |
|  | Functional connections | - | VLMT-inter | 77 |
|  | Functional connections | - | VLMT-6^th^ | 77 |
|  | Functional connections | - | VLMT-7^th^ | 77 |
|  | Functional connections | - | VLMT-recog | 76 |
|  | Functional connections | - | ERT | 76 |
|  |  |  |  |  |
| Mediation analysis | Aerobic fitness | Functional connections | PANSS-positive | 58 |
|  | Aerobic fitness | Functional connections | PANSS-negative | 57 |
|  | Aerobic fitness | Functional connections | PANSS-psychopath | 58 |
|  | Aerobic fitness | Functional connections | PANSS-total | 58 |
|  | Aerobic fitness | Functional connections | CDSS | 58 |
|  | Aerobic fitness | Functional connections | CGI | 58 |
|  | Aerobic fitness | Functional connections | B-CATS-animals | 55 |
|  | Aerobic fitness | Functional connections | B-CATS-fruits | 55 |
|  | Aerobic fitness | Functional connections | B-CATS-vegetables | 51 |
|  | Aerobic fitness | Functional connections | DSST | 56 |
|  | Aerobic fitness | Functional connections | DST-backward | 57 |
|  | Aerobic fitness | Functional connections | TMT-A | 57 |
|  | Aerobic fitness | Functional connections | TMT-B | 56 |
|  | Aerobic fitness | Functional connections | VLMT-sum | 56 |
|  | Aerobic fitness | Functional connections | VLMT-inter | 56 |
|  | Aerobic fitness | Functional connections | ERT | 55 |

*Note.* For each statistical approach the final sample size is shown. In case of the clinical and cognitive data, sample sizes vary depending on the corresponding test battery. With respect to the mediation analysis, only those clinical and cognitive tests were considered that showed correlations with certain functional connections. The number of investigated functional connections did not differ between subjects.

**S7: Parameters in Bayesian statistics**

A narrow stretched beta distribution (κ = 0.196) was utilized as the prior within the multilevel partial correlation design because possible effect sizes were expected to be small [18]. Aiming at evaluating the presence of an association between aerobic fitness and the corresponding variable of interest, the following statistical parameters were considered within the Bayesian framework:

1. Jeffrey´s default Bayes Factor (BF_10_): the probability odds between the alternative and the null hypothesis. A BF_10_ = 3 means that it is three times more likely to observe the data given the alternative hypothesis than under the null hypthesis [19]. Table S7 displays a common interpretation scheme according to Lee and Wagenmakers [20] adjusted from Jeffreys [21].
2. Pearson´s correlation coefficient (r_p_): the strength of the linear relation between two variables.
3. Highest Density Interval (HDI): the range in which the true correlation coefficient falls with a probability of 89%.
4. Probability of direction (PD): the probability in percent that the correlation goes in one direction (positive vs. negative correlation coefficients). If r = 0.3 and PD = 99 %, the probability of the correlation coefficient to be positive equals 99 %. The larger PD, the more likely is an existing association between two variables.
5. Region of practical equivalence (ROPE): overlap in percent between the HDI and a predefined area around zero ranging from -0.1 to 0.1. If ROPE = 2 %, the overlap between the HDI and the predefined area equals 2 % indicating that the true correlation coefficient is very likely to be larger than 0.1 or smaller than -0.1. The smaller ROPE, the more likely is an existing association between two variables.

**Tab. S7**

Interpretation of the BF

| Bayes factor. ${BF}_{10}$ | Interpretation |
| --- | --- |
| > 100 | Extreme evidence for $H_{1}$compared to $H_{0}$ |
| 30 – 100 | Very strong evidence for $H_{1}$compared to $H_{0}$ |
| 10 – 30 | Strong evidence for $H_{1}$compared to $H_{0}$ |
| 3 – 10 | Moderate evidence for $H_{1}$compared to $H_{0}$ |
| 1 – 3 | Anecdotal evidence for $H_{1}$compared to $H_{0}$ |
| 1 | No evidence in favour of $H_{0}$ or $H_{1}$ |
| 1/3 – 1 | Anecdotal evidence for $H_{0}$compared to $H_{1}$ |
| 1/10 – 1/3 | Moderate evidence for $H_{0}$compared to $H_{1}$ |
| 1/30 – 1/10 | Strong evidence for $H_{0}$compared to $H_{1}$ |
| 1/100 – 1/30 | Very strong evidence for $H_{0}$compared to $H_{1}$ |
| < 1/100 | Extreme evidence for $H_{0}$compared to $H_{1}$ |

*Note.* This table displays an interpretation scheme of the BF according to Lee and Wagenmakers [20] adjusted from Jeffreys [21]. ${BF}_{10}$ means that the likelihood of the data under $H_{1}$is compared to the likelihood of the data under $H_{0}$.

**S8: Definition of anatomical clusters**

The 6670 functional connections between all AAL-regions in the seed-based approach were assigned to 45 anatomical clusters based on the anatomical description proposed by Tzourio-Mazoyer et al. [22]. For instance, the functional connection between the right middle frontal gyrus and the right thalamus was assigned to belong to the FRONT-NUC cluster because the middle frontal gyrus is part of the frontal lobe, while the thalamus belongs to the subcortical nuclei. Table S8 illustrates the meanings of the cluster abbreviations. We performed this procedure with all 6670 single connections that were linked to AF. Thereafter, we examined if certain anatomical clusters were linked more robustly to AF than others. In order to evaluate robustness, we considered the proportion of functional connections correlated with AF within each cluster, the consistency of the direction of the correlations and the strength of evidence across correlation tests. The documentation sheet of these three criteria including our final decisions and the results from the partial correlation tests in the seed-based approach will be published on OSF. If an anatomical cluster comprised multiple functional connections with the majority of the correlations to AF going in the same direction accompanied by high BFs, the corresponding cluster was supposed to be robust.

**Tab. S8**

Assignment of regions to clusters.

| abbreviations | regions |
| --- | --- |
| NUC | subcortical nuclei (thalamus, amygdala, putamen, pallidum, caudate) |
| CEREB | Seeds of the cerebellum |
| CENTRAL | Central regions (Postcentral gyrus, precentral gyrus, rolandic operculum) |
| LIM | Limbic lobe (temporal pole, anterior cingulate gyrus, median cingulate gyrus, posterior cingulate gyrus, hippocampus, parahippocampal gyrus) |
| TEMP | Temporal lobe (superior, middle and inferior temporal gyrus, Heschl gyrus) |
| FRONT | Frontal lobe (superior, middle and inferior frontal gyrus, orbital and medial parts of the superior, middle and inferior frontal gyrus, supplementary motor area, paracentral lobule, gyrus rectus, olfactory cortex) |
| PARIETAL | Parietal lobe (superior and inferior parietal gyrus, angular gyrus, supramarginal gyrus, precuneus) |
| OCC | Occipital lobe (superior, middle and inferior occipital lobe, cuneus, calcarine fissure, lingual gyrus and fusiform gyrus) |
| INS | insula |

*Note.* List of regions comprised by the abbreviations based on AAL-atlas [22]. The combination of these abbreviations builds an anatomical cluster.

**S9: Bayes Factor Design Analysis (BFDA)**

Figure S9 shows the distribution of the BFs after Monte Carlo simulations of 10000 hypothetical studies examining the correlation between AF and FC within a fixed-N design given the alternative and given the null hypothesis. The following parameters were set in order to perform the BFDA:

1. Effect size under alternative hypothesis: r = N(0.2.0.1²)
2. Effect size under null hypothesis: r = N(0.0.03²)
3. Prior: Beta(κ = 0.196)
4. Sample size: n = 58
5. Alternative = two-tailed

Given the alternative hypothesis, the probability to observe a BF higher than three was 30.6 %, whereas in 69.4 % of the cases the BF was lower. Given the null hypothesis, in 8.4 % of the cases the BF was higher than three, while it was lower in 91.6 % of the cases. Under the aforementioned assumptions, the probability of false negatives was much higher than the probability of false positives for the analysis of the fMRI data.

**Fig. S9**

BFDA regarding the associations between aerobic fitness and the whole-brain functional connections

*Note.* Distributions of the BFs resulting from Monte Carlo simulations of 10000 hypothetical studies within a fixed-N design given the alternative and given the null hypothesis.

**S10: Correlations between FC and clinical and cognitive scores in seed-based approach**

In order to ensure the clarity of the main manuscript, the results from the multilevel Bayesian partial correlations between functional connections from the eight anatomical clusters related to AF and clinical and cognitive outcome are visualized in this supplemental section. Figures S10.1 – S10.8 illustrate the findings.

**Fig. S10.1**

BFs and partial correlations between functional connections from the CENTRAL-CENTRAL cluster and clinical and cognitive scores

*Note.* Visualization of the BFs and correlation coefficients of the Bayesian multilevel partial correlation tests including functional connections from the CENTRAL-CENTRAL cluster and clinical and cognitive scores. Correlation tests resulting in a BF_10_ around three or higher are labelled with the corresponding name of the test battery.

**Fig. S10.2**

BFs and partial correlations between functional connections from the CENTRAL-LIM cluster and clinical and cognitive scores

*Note.* Visualization of the BFs and correlation coefficients of the Bayesian multilevel partial correlation tests including functional connections from the CENTRAL-LIM cluster and clinical and cognitive scores. Correlation tests resulting in a BF_10_ around three or higher are labelled with the corresponding name of the test battery.

**Fig. S10.3**

BFs and partial correlations between functional connections from the CEREB-CEREB cluster and clinical and cognitive scores

*Note.* Visualization of the BFs and correlation coefficients of the Bayesian multilevel partial correlation tests including functional connections from the CEREB-CEREB cluster and clinical and cognitive scores. Correlation tests resulting in a BF_10_ around three or higher are labelled with the corresponding name of the test battery.

**Fig. S10.4**

BFs and partial correlations between functional connections from the CEREB-NUC cluster and clinical and cognitive scores

*Note.* Visualization of the BFs and correlation coefficients of the Bayesian multilevel partial correlation tests including functional connections from the CEREB-NUC cluster and clinical and cognitive scores. Correlation tests resulting in a BF_10_ around three or higher are labelled with the corresponding name of the test battery.

**Fig. S10.5**

BFs and partial correlations between functional connections from the LIM-TEMP cluster and clinical and cognitive scores

*Note.* Visualization of the BFs and correlation coefficients of the Bayesian multilevel partial correlation tests including functional connections from the LIM-TEMP cluster and clinical and cognitive scores. Correlation tests resulting in a BF_10_ around three or higher are labelled with the corresponding name of the test battery.

**Fig. S10.6**

BFs and partial correlations between functional connections from the NUC-NUC cluster and clinical and cognitive scores

*Note.* Visualization of the BFs and correlation coefficients of the Bayesian multilevel partial correlation tests including functional connections from the NUC-NUC cluster and clinical and cognitive scores. Correlation tests resulting in a BF_10_ around three or higher are labelled with the corresponding name of the test battery.

**Fig. S10.7**

BFs and partial correlations between functional connections from the OCC-TEMP cluster and clinical and cognitive scores

*Note.* Visualization of the BFs and correlation coefficients of the Bayesian multilevel partial correlation tests including functional connections from the OCC-TEMP cluster and clinical and cognitive scores. Correlation tests resulting in a BF_10_ around three or higher are labelled with the corresponding name of the test battery.

**Fig. S10.8**

BFs and partial correlations between functional connections from the TEMP-TEMP cluster and clinical and cognitive scores

*Note.* Visualization of the BFs and correlation coefficients of the Bayesian multilevel partial correlation tests including functional connections from the TEMP cluster and clinical and cognitive scores. Correlation tests resulting in a BF_10_ around three or higher are labelled with the corresponding name of the test battery.

**Literature**

1. Tustison NJ, Avants BB, Cook PA, Zheng Y, Egan A, Yushkevich PA, Gee JC (2010) N4itk: Improved n3 bias correction. IEEE Trans Med Imaging 29:1310-1320. <https://doi.org/10.1109/tmi.2010.2046908>

2. Avants BB, Tustison N, Song G (2009) Advanced normalization tools (ants). Insight j 2:1-35.

3. Fonov VS, Evans AC, McKinstry RC, Almli CR, Collins DL (2009) Unbiased nonlinear average age-appropriate brain templates from birth to adulthood. NeuroImage 47:102. <https://doi.org/10.1016/S1053-8119(09)70884-5>

4. Avants BB, Epstein CL, Grossman M, Gee JC (2008) Symmetric diffeomorphic image registration with cross-correlation: Evaluating automated labeling of elderly and neurodegenerative brain. Med Image Anal 12:26-41. <https://doi.org/10.1016/j.media.2007.06.004>

5. Zhang Y, Brady M, Smith S (2001) Segmentation of brain mr images through a hidden markov random field model and the expectation-maximization algorithm. IEEE Transactions on Medical Imaging 20:45-57. <https://doi.org/10.1109/42.906424>

6. Jenkinson M, Beckmann CF, Behrens TE, Woolrich MW, Smith SM (2012) Fsl. NeuroImage 62:782-790. <https://doi.org/10.1016/j.neuroimage.2011.09.015>

7. Cox RW (1996) Afni: Software for analysis and visualization of functional magnetic resonance neuroimages. Computers and Biomedical Research 29:162-173. <https://doi.org/https://doi.org/10.1006/cbmr.1996.0014>

8. Jenkinson M, Bannister P, Brady M, Smith S (2002) Improved optimization for the robust and accurate linear registration and motion correction of brain images. NeuroImage 17:825-841. <https://doi.org/https://doi.org/10.1006/nimg.2002.1132>

9. Wang S, Peterson DJ, Gatenby JC, Li W, Grabowski TJ, Madhyastha TM (2017) Evaluation of field map and nonlinear registration methods for correction of susceptibility artifacts in diffusion mri. Front Neuroinform 11:17. <https://doi.org/10.3389/fninf.2017.00017>

10. Huntenburg JM, Gorgolewski KJ, Anwander A, Margulies DS (2014) Evaluating nonlinear coregistration of bold epi and t1 images. F1000 Res 5:740.

11. Treiber JM, White NS, Steed TC, Bartsch H, Holland D, Farid N, McDonald CR, Carter BS, Dale AM, Chen CC (2016) Characterization and correction of geometric distortions in 814 diffusion weighted images. PLOS ONE 11:e0152472. <https://doi.org/10.1371/journal.pone.0152472>

12. Greve DN, Fischl B (2009) Accurate and robust brain image alignment using boundary-based registration. NeuroImage 48:63-72. <https://doi.org/10.1016/j.neuroimage.2009.06.060>

13. Jenkinson M, Smith S (2001) A global optimisation method for robust affine registration of brain images. Med Image Anal 5:143-156. <https://doi.org/10.1016/s1361-8415(01)00036-6>

14. Power JD, Mitra A, Laumann TO, Snyder AZ, Schlaggar BL, Petersen SE (2014) Methods to detect, characterize, and remove motion artifact in resting state fmri. NeuroImage 84:320-341. <https://doi.org/10.1016/j.neuroimage.2013.08.048>

15. Gorgolewski K, Burns CD, Madison C, Clark D, Halchenko YO, Waskom ML, Ghosh SS (2011) Nipype: A flexible, lightweight and extensible neuroimaging data processing framework in python. Front Neuroinform 5:13. <https://doi.org/10.3389/fninf.2011.00013>

16. Pruim RHR, Mennes M, van Rooij D, Llera A, Buitelaar JK, Beckmann CF (2015) Ica-aroma: A robust ica-based strategy for removing motion artifacts from fmri data. NeuroImage 112:267-277. <https://doi.org/10.1016/j.neuroimage.2015.02.064>

17. Laird AR, Fox PM, Eickhoff SB, Turner JA, Ray KL, McKay DR, Glahn DC, Beckmann CF, Smith SM, Fox PT (2011) Behavioral interpretations of intrinsic connectivity networks. J Cogn Neurosci 23:4022-4037. <https://doi.org/10.1162/jocn_a_00077>

18. Rouder JN, Speckman PL, Sun D, Morey RD, Iverson G (2009) Bayesian t tests for accepting and rejecting the null hypothesis. Psychon Bull Rev 16:225-237. <https://doi.org/10.3758/pbr.16.2.225>

19. Ly A, Verhagen J, Wagenmakers E-J (2016) Harold jeffreys’s default bayes factor hypothesis tests: Explanation, extension, and application in psychology. J Math Psychol 72:19-32. <https://doi.org/10.1016/j.jmp.2015.06.004>

20. Lee MD, Wagenmakers E-J (2013) Bayesian cognitive modeling: A practical course. Cambridge University Press, Cambridge

21. Jeffreys H (1998) The theory of probability. Oxford Univerity Press, Oxford

22. Tzourio-Mazoyer N, Landeau B, Papathanassiou D, Crivello F, Etard O, Delcroix N, Mazoyer B, Joliot M (2002) Automated anatomical labeling of activations in spm using a macroscopic anatomical parcellation of the mni mri single-subject brain. NeuroImage 15:273-289. <https://doi.org/10.1006/nimg.2001.0978>
